# Polydimethylsiloxanes with Grafted Dibenzoylmethanatoboron Difluoride: Synthesis and Properties

**DOI:** 10.3390/polym14235075

**Published:** 2022-11-23

**Authors:** Anastasia S. Belova, Arevik G. Khchoyan, Tatiana M. Il’ina, Yuriy N. Kononevich, Dmitry S. Ionov, Viacheslav A. Sazhnikov, Dmitry A. Khanin, Galina G. Nikiforova, Viktor G. Vasil’ev, Aziz M. Muzafarov

**Affiliations:** 1 A.N. Nesmeyanov Institute of Organoelement Compounds of Russian Academy of Sciences, 119991 Moscow, Russia; 2 Faculty of Chemical-Pharmaceutical Technologies and Biomedical Preparations, D. Mendeleev University of Chemical Technology of Russia, 125047 Moscow, Russia; 3 Faculty of Technology of Inorganic Substances and High-Temperature Materials, D. Mendeleev University of Chemical Technology of Russia, 125047 Moscow, Russia; 4 Photochemistry Center, FSRC “Crystallography and Photonics” of Russian Academy of Sciences, 1119421 Moscow, Russia; 5 N.S. Enikolopov Institute of Synthetic Polymeric Materials of Russian Academy of Sciences, 117393 Moscow, Russia

**Keywords:** dibenzoylmethanatoboron difluoride, polydimethylsiloxane, H-dimer, excimer

## Abstract

A method for the preparation of polydimethylsiloxanes with grafted methoxy-substituted dibenzoylmethanatoboron difluoride has been described. The structures of prepared polymers were confirmed using NMR, IR spectroscopy and gel permeation chromatography methods. Their thermal properties were investigated using thermal gravimetric analysis, differential scanning calorimetry and thermomechanical analysis. The prepared polymers had good thermal (T_d_^5%^ up to 393 °C) and thermo-oxidative (T_d_^5%^ = 413 °C) stability. The polymers started to transit in a viscous flow state at about 40 °C (for **3 a**) and at about 20 °C (for **3 b**). The viscoelastic characteristics of prepared polymers were determined in the sinusoidal oscillating vibrations mode. It was shown that the studied polymers at low frequencies at room temperature are viscoelastic fluids (G′ < G″). Increasing the frequency led to inversion (crossover) of dependences G′ and G″, which indicated the transition of polymers from viscous to elastomeric behavior characteristics, and the beginning of the formation of a physical network. Optical properties were studied using electron absorption, steady-state and time-resolved fluorescence spectroscopy. It was shown that intramolecular H-dimers exist in the ground state. The polymers studied had a bright fluorescence in the solution and in the solid state, consisting of bands of monomer and excimer emission. Thermally-activated delayed fluorescence was observed in the solution and the solid state. The prepared polymers possess intriguing properties that make them useful as optical materials, sensors or imaging agents.

## 1. Introduction

Currently, fluorescent polymers are of increasing interest for their potential applications in various fields of science and technology such as organic electronics, sensing, imaging, biomedical applications, etc. There are two routes by which a fluorophore is introduced into the polymer matrix: mixing with a polymer, and covalent linking. Despite the simplicity of fluorophore incorporation into the polymer matrix by mixing, this method has obvious disadvantages; the main ones are related to a phase separation in the matrix and a leaching of the fluorophore from the polymeric matrix. The second approach, based on covalent attachment, allows these possible problems to be avoided.

Among a great variety of polymers, one of the most promising categories is polysiloxanes because of their significant properties such as high elasticity and flexibility, high hydrophobicity, good thermal stability, tunable mechanical properties, biocompatibility and low toxicity, superior film-forming ability, and resistance to thermal, chemical, and radiation degradation [1]. Such properties as tunable refractive index, optical transparency and excellent photostability make polysiloxanes very attractive for numerous optoelectronic applications [2,3]. The combination of polysiloxane with fluorophores leads to fluorescent polymers with unique properties which allow them to find numerous applications as materials for organic light emitting diodes, pressure-sensitive polymers, fluorescent stress-strain sensors, chemosensors, etc. There are many examples of fluorophores being introduced into the linear polysiloxane matrix in terminal position, grafted to the main chain or integrated directly into the main chain. As an example, polydimethylsiloxane (PDMS) with terminal pyrene fragments was used for the study of temperature-dependent tail-tail dynamics in polymers [4]. Pyrene-labelled polydimethylsiloxane networks were used for the noninvasive in situ monitoring of stress and strain in silicone elastomers [5]. PDMS containing coumarin co-polymers was proposed for use as a sensing material for the detection of nitroaromatics-containing explosives [6]. Due to the simplicity of their synthesis, polysiloxanes with grafted fluorophores are widespread [7]. Cross-linking of fluorophore-containing polysiloxanes leads to elastomeric materials with mechanosensitive Förster resonance energy transfer (FRET) [8]. Along with organic fluorophores, inorganic luminescent complexes are also used in combination with polysiloxane matrices. Thus, luminescent polysiloxanes can possess self-healing properties [9,10,11,12], be applied in photonics [13,14,15], and as sensing devices for detecting nitroaromatic compounds [16], oxygen [17,18] or ammonia [9]. There are many examples of luminescent silicone materials based on lanthanide complexes with numerous ligands [19,20,21,22].

It is well known that boron-containing fluorescent complexes, such as derivatives of boron dipyrromethene (BODIPY), dibenzoylmethanatoboron difluoride (DBMBF_2_) and others, possess impressive photophysical/photochemical properties and are promising objects in the development of new fluorescent materials for organic electronics and photonics [23,24,25]. These fluorophores can find application as optical [26,27], mechanochromic [28,29,30,31,32,33,34,35,36], or photochromic [37,38] materials, as well as sensors for the detection of oxygen [39], amines [40], and aromatic compounds [41,42,43]. Incorporation of these complexes into the polymer matrix with the next study of properties is a current trend in polymer chemistry and materials science [44,45]. It should be mentioned that much attention has been given in the literature to polymers containing boron dipyrromethene (BODIPY) derivatives due to their outstanding photophysical properties such as a high extinction coefficient, sharp emission bands, a high fluorescence quantum yield, large two-photon absorption cross section, negligible formation of triplet states, and excellent chemical and thermal stability. Thus, BODIPY derivative-containing fluorescent thermo-sensitive polymers [46,47], ultrabright fluorescent polymer nanoparticles [48], labeled polyolefins [49], fluorescent polymer brushes [50], etc. were prepared and studied. Meanwhile, DBMBF_2_-containing polymers are less described in the literature and are being systematically studied by the Fraser, Zhang, and Mirochnik groups. Accordingly, methyl methacrylate and styrene polymers (copolymers) containing BF_2_-benzoylacetonate (BF_2_-BAc) groups [51,52] and polystyrene with difluoroboron avobenzone complexes at side chains [53] were prepared and studied. By incorporating DBMBF_2_ into poly(lactic acid), poly(ε-caprolactone)—a single-component, multi-emissive material exhibiting intense fluorescence, delayed fluorescence, and unusual room-temperature phosphorescence—was obtained [54,55,56]. There are several examples of polymer compositions based on DBMBF_2_ derivatives without covalent linking of the fluorophore to the polymer matrix. Thus, the photochemical behavior of DBMBF_2_ derivatives incorporated in the polyethylene, polystyrene, polymethyl methacrylate and polyacrylate matrix by mixing was studied [57,58,59,60]. To the best of our knowledge, regarding the study of boron-containing polysiloxanes, this area has not been studied enough and only single examples such as PDMS with grafted BODIPY derivatives [7], or DBMBF_2_-containing polysiloxanes [61] are known.

Continuing our study of the behavior of DBMBF_2_ derivatives connected through flexible siloxane linker [62,63], in the present work the method for the preparation and properties of polydimethylsiloxanes with grafted dibenzoylmethanatoboron difluoride has been described for the first time. The structures of the prepared polymers were confirmed using NMR, IR spectroscopy and gel permeation chromatography (GPC) methods. The thermal properties were investigated by thermal gravimetric analysis (TGA), differential scanning calorimetry (DSC) and thermomechanical analysis (TMA). The rheological properties of the prepared polymers were investigated in the sinusoidal oscillating vibrations mode. Their optical properties were studied using electron absorption, steady-state and time-resolved fluorescence spectroscopy. The prepared polymers possess intriguing properties that make them useful as optical materials, sensors, imaging agents, etc.

## 2. Experimental

### 2.1. Materials

Toluene was distilled from CaH_2_. Platinum(0)-1,3-divinyl-1,1,3,3-tetramethyldisiloxane complex (Karstedt’s catalyst) solution (in xylene, Pt-2%) was purchased from Sigma-Aldrich. Allyl-DBMBF_2_-OMe **2** was prepared using the method described by us in a previous publication [63].

#### 2.1.1. Synthesis of Polysiloxane with Distributed SiH Groups (**1 a,b**)

Polysiloxanes with distributed silylhydride groups **1 a,b** were prepared using the method described earlier by us [9].

**1 a**: **M_n_** = 31,800 Da, **M_w_** = 47,000 Da, **PDI** = 1.5. **^1^H NMR** (400 MHz, CDCl_3_): *δ* 0.07 (s, 173 H, Si-CH_3_), 4.68 (m, 1H, Si-H). **^13^C NMR** (101 MHz, CDCl_3_): *δ* 1.4, 1.3, 1.0, 0.9, 0.8, 0.6. **^29^Si NMR** (79 MHz, CDCl_3_): *δ* −20.5, −21.6, −21.9. **IR** (KBr, cm^–1^): 2964, 2906, 2157, 1413, 1262, 1094, 1024, 913, 865, 801, 704.

**1 b**: **M_n_** = 30,800 Da, **M_w_** = 47,200 Da, **PDI** = 1.5. **^1^H NMR** (400 MHz, CDCl_3_): *δ* 0.07 (s, 1240 H, Si-CH_3_), 4.68 (m, 1H, Si-H). **^13^C NMR** (101 MHz, CDCl_3_): *δ* 1.0. **^29^Si NMR** (79 MHz, CDCl_3_): *δ* −21.9. **IR** (KBr, cm^–1^): 2964, 2906, 2156, 1413, 1262, 1095, 1022, 865, 801, 704.

#### 2.1.2. Synthesis of Polysiloxane with Distributed DBMBF_2_ Groups (**3 a,b**)

A mixture of polysiloxane **1 a,b** (5 g), allyl-DBMBF_2_-OMe **2** (1.37 g, 4 mmol in the case of **1 a**; 0.137 g, 0.4 mmol in the case of **1 b**) and 50 μL solution of Karstedt’s catalyst was stirred in dry toluene (50 mL) under argon atmosphere at 40 °C for 48 h. After the reaction was complete, the solvent was removed by rotor evaporator and residue was purified using preparative gel-permission chromatography. Polymers **3 a,b** were obtained as yellow elastic solids.

**3 a**. **Yield**: 25%. **M_n_** = 49,400 Da, **M_w_** = 453,100 Da, **PDI** = 9.2. **IR** (KBr, cm^–1^): 2963, 2906, 1607, 1551, 1501, 1444, 1414, 1375, 1315, 1261, 1178, 1093, 1019, 864, 798, 703, 662, 554. **^1^H NMR** (400 MHz, CDCl_3_): *δ* 0.07 (s, 172H, SiCH_3_), 0.56 (m, 2H, SiCH_2_), 1.70 (m, 2H, CH_2_), 2.71 (m, 2H, CH_2_), 3.92 (s, 3H, OCH_3_), 7.01 (m, 2H, Ar), 7.06 (s, 1H, COCHCO), 7.31 (m, 2H, Ar), 8.04 (m, 2H, Ar), 8.13 (m, 2H, Ar). **^13^C NMR** (101 MHz, CDCl_3_) *δ* 181.6, 181.5, 165.5, 150.9, 131.5, 129.8, 129.2, 128.8, 124.2, 114.6, 92.1, 55.8, 39.6, 24.7, 17.2, 1.4, 1.1, 1.0, 0.7, −0.5. **^29^Si NMR** (79 MHz, CDCl_3_): *δ* −21.91.

**3 b**. **Yield**: 80%. **M_n_** = 64,500 Da, **M_w_** = 244,100 Da, **PDI** = 3.8. **IR** (KBr, cm^–1^): 2963, 2906, 1607, 1550, 1501, 1445, 1413, 1376, 1261, 1092, 1018, 865, 800, 703, 662. **^1^H NMR** (400 MHz, CDCl_3_): *δ* 0.07 (s, 1240 H, Si-CH_3_), 0.56 (m, 2H, SiCH_2_), 1.71 (m, 2H, CH_2_), 2.73 (m, 2H, CH_2_), 3.93 (s, 3H, OCH_3_), 7.03 (d, 2H, *J* = 8.5 Hz, Ar), 7.08 (s, 1H, COCHCO), 7.33 (m, 2H, *J* = 6.0 Hz, Ar), 8.05 (m, 2H, *J* = 8.2 Hz, Ar), 8.15 (m, 2H, *J* = 8.8 Hz, Ar). **^13^C NMR** (101 MHz, CDCl_3_) *δ* 0.65, 1.02, 1.39. **^29^Si NMR** (79 MHz, CDCl_3_): *δ* −21.93.

### 2.2. Characterization

^1^H, ^13^C and ^29^Si NMR spectra were recorded on a Bruker Avance II spectrometer (400 MHz; Bruker, Ettlingen, Germany). Chemical shifts were reported relative to chloroform (δ = 7.25 ppm) for ^1^H NMR. IR spectra were recorded on an IR spectrometer with a Fourier transformer Shimadzu IRTracer-100 (Shimadzu, Kyoto, Japan). KBr pellets were used as samples. Gel permeation chromatography (GPC) analyses were performed in toluene and THF (1 mL/min) using a Shimadzu Prominent system (Shimadzu, Kyoto, Japan) equipped with RID-20A refractive index detector. The GPC columns (Phenogel) were calibrated with polystyrene standards (PSS). Thermogravimetric analysis (TGA) was performed using Shimadzu DTG-60H simultaneous TG/DTA thermal analyzer (Shimadzu, Kyoto, Japan) on samples weighing about 5 mg at a heating rate of 10 °C/min in air and argon. The temperature at which a weight loss of 5% was detected was considered to be the decomposition onset temperature. Differential scanning calorimetry (DSC) analysis was performed using a DSC-3 Mettler Toledo differential scanning calorimeter (Greifensee, Switzerland) at a heating rate of 10 °C/min in argon. Thermomechanical analysis (TMA) was performed using a thermal mechanical analyzer TMA/SDTA 2 + LN/600 from Mettler Toledo (Greifensee, Switzerland) at a heating rate of 5 °C/min. Gel fraction analysis was performed through the extraction of polymers with chloroform for 72 h in a Soxhlet apparatus. Finally, the samples were dried in a vacuum oven at 1 mBar and 80 °C until a constant weight was reached. The gel fractions in the samples were calculated using the following equation:

Gel fraction (%) = W_1_/W_0_ × 100

where W_0_ and W_1_ are the dried-gel weights before and after the extraction, respectively. The absorption spectra were recorded on a Shimadzu UV-1900 spectrophotometer (Shimadzu, Kyoto, Japan). The fluorescence spectra were acquired using a Shimadzu RF-6000 spectrofluorophotometer (Shimadzu, Kyoto, Japan). Spectroscopic grade solvents (Aldrich, St. Louis, MO, USA) were used in UV-vis absorption and fluorescence measurements. The quantum yields of the fluorescence of studied compounds were measured using deaerated solutions. A solution of 9,10-diphenylanthracene in cyclohexane purged with argon (Φ_f_ = 0.9) was used as the fluorescence standard for calculating the quantum yield. Fluorescence decay curves and delayed fluorescence spectra were acquired on a Fluotime 300 spectrofluorometer (Picoquant, Berlin, Germany). An LDH-D-C-375 laser was used as the excitation source (λ_ex_ = 375 nm). Data fitting was performed with Easytau2 (Picoquant) software using multiexponential fitting of the experimental data:

$$\mathrm{Dec}\left(t\right)=\left[\int_{-\infty }^{t}dt'\left[IRF\left(t-Shift_{IRF}\right)-Bkgr_{IRF}\right]\left[\overset{n_{Exp}}{\underset{i=1}{\sum }}A_{i}e^{-\frac{t-t^{\prime }}{\tau_{i}}}+A_{Scatt}\delta \left(t-t^{\prime }\right)\right]\right]+Bkgr_{Dec}$$

$$I_{m}=A_{m}\tau_{m}$$

$$\tau_{Av\;Int}=\overset{n_{exp}}{\underset{\begin{array}{c} i=1\\ I_{i}>0 \end{array}}{\sum }}I_{i}\tau_{i}/\overset{n_{exp}}{\underset{\begin{array}{c} i=1\\ I_{i}>0 \end{array}}{\sum }}I_{i}$$

$$\tau_{Av\;Amp}=\overset{n_{exp}}{\underset{\begin{array}{c} i=1\\ A_{i}>0 \end{array}}{\sum }}A_{i}\tau_{i}/\overset{n_{exp}}{\underset{\begin{array}{c} i=1\\ A_{i}>0 \end{array}}{\sum }}A_{i}$$

When measuring the dependence of fluorescence decay curves on the detection wavelength, two sets of data were obtained, on account of the significant difference between lifetimes and intensities of fluorophore and its excimers. The first dataset was measured in the range of 380–500 nm with frequency 40 MHz, and the second in the range of 500–700 nm with frequency 1–5 MHz. The excitation and emission attenuators were differently optimized when these two datasets were obtained. Time-resolved spectra with the same measurement parameters for all wavelengths were obtained for solid state samples of polymers **3 a,b**, and solution of polymer **3 a** in cyclohexane. Simultaneous global fitting was performed for the obtained data due to the consideration that fluorescence lifetimes can be comparable or higher than the excitation period in the first dataset. The excitation period was fixed for each measurement according to the excitation frequency. To choose an appropriate number of exponential terms, we used the criteria that χ^2^ was in the range of 0.8–1.3 and the uniformity of residuals distribution and auto-correlation function was around zero. The FWHM of the instrument response function (IRF) was about 180 ps.

## 3. Results and Discussion

### 3.1. Preparation

**Scheme 1:**
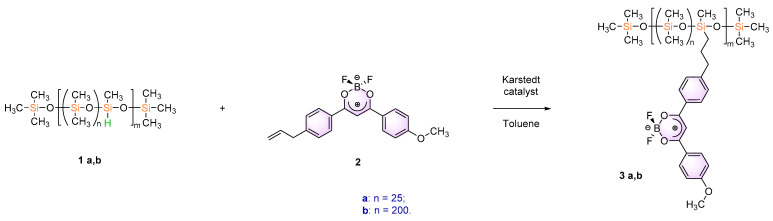
Preparation of DBMBF_2_-grafted polydimethylsiloxanes **3 a,b**.

**Figure 1:**
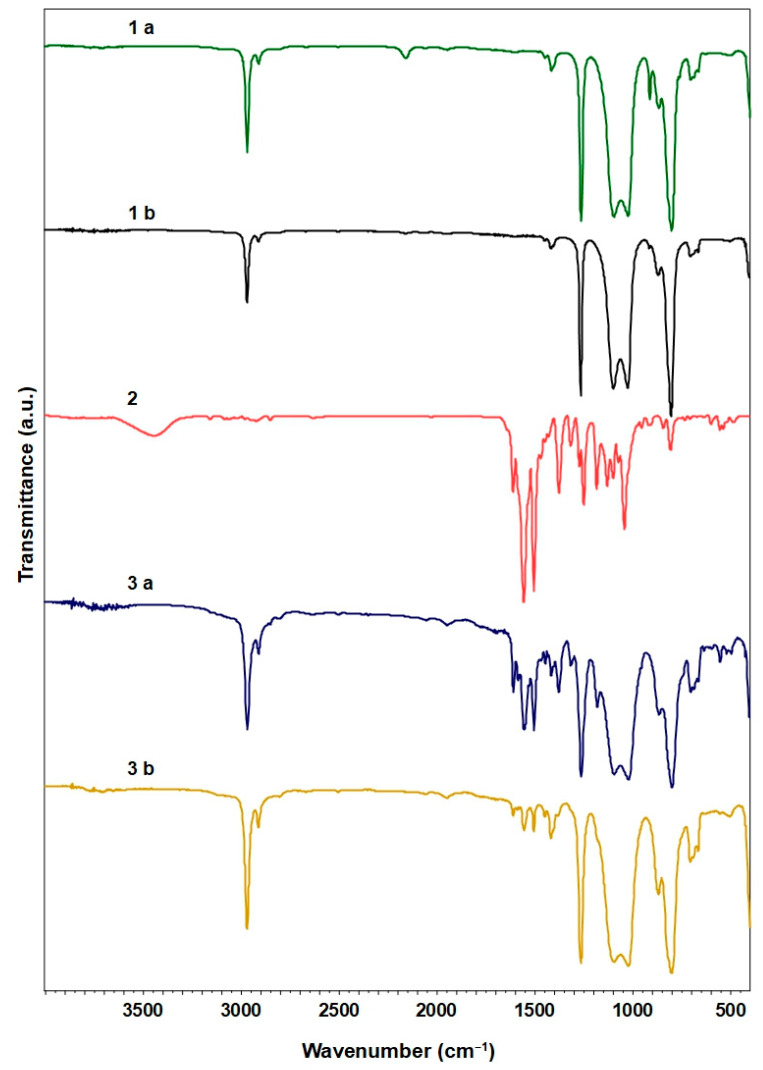
FTIR spectra of **1 a,b**, **2**, and **3 a,b**.

Polydimethylsiloxanes (PDMSs) with grafted dibenzoylmethanatoboron difluoride (DBMBF_2_) were prepared according to Scheme 1. Initial PDMSs with distributed silylhydride groups **1 a,b** were synthesized using the method described earlier by us [9] and had the following molecular characteristics: **1 a**: M_n_ = 31,800 Da, M_w_ = 47,000 Da, PDI = 1.5. **1 b**: M_n_ = 30,800 Da, M_w_ = 47,200 Da, PDI = 1.5. GPC curves of polymers **1 a,b** obtained in toluene are presented in Figures S1 and S2 (Supporting Information). ^1^H NMR spectra of **1 a,b** contained signals that were assigned to protons of Si-CH_3_ groups (singlet at 0.07 ppm) and protons of silylhydride groups (multiplet at 4.68 ppm) and are presented in Figures S3 and S6 (Supporting Information (SI)). An integration of signals in these spectra gives a ratio of distributed chains -O-SiHCH_3_- to -O-Si(CH_3_)_2_- as 1:25 for **1 a** and 1:200 for **1 b**. The FTIR spectra of **1 a,b** presented in Figure 1 show characteristic absorption peaks at 1022–1095 cm^−1^ and 801 cm^−1^ that are assigned to asymmetric and symmetric Si-O-Si stretching vibrations, correspondingly. The peaks at 1262 and 865 cm^−1^ are attributed to the Si-C vibrations, and peaks at 2906–2964 cm^−1^ are attributed to the C-H vibrations. The low-intensity absorption band at 2156 cm^−1^ is assigned to the Si-H stretching vibration. Functional allyl-DBMBF_2_-OMe **2** was prepared using the method described earlier by us [63].

Using the hydrosilylation reaction between polysiloxane **1 a,b** containing distributed silylhydride groups and allyl-DBMBF_2_-OMe **2** in toluene in the presence of Karstedt’s catalyst, polysiloxanes **3 a,b** with grafted DBMBF_2_ groups have been synthesized as yellow elastic solids (Scheme 1). The products **3 a,b** were purified through gel permeation chromatography using THF as an eluent.

**Figure 2:**
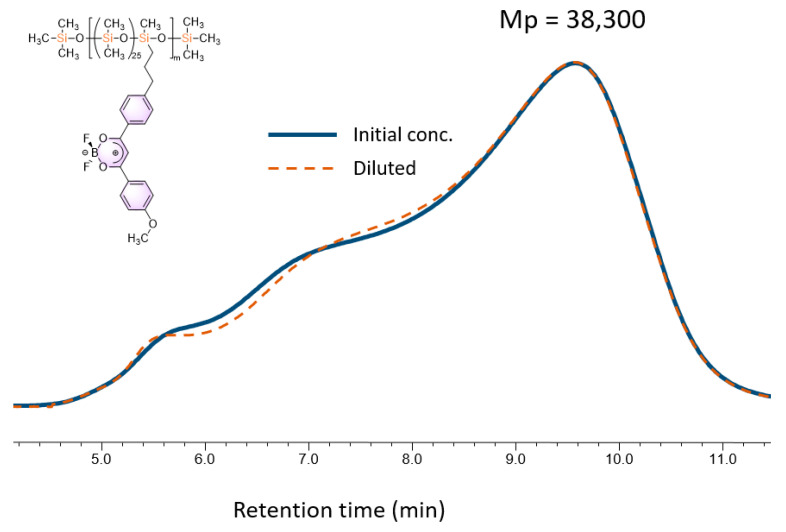
GPC curves of polymer **3 a** obtained in THF using column Phenogel 10^5^ Å.

**Figure 3:**
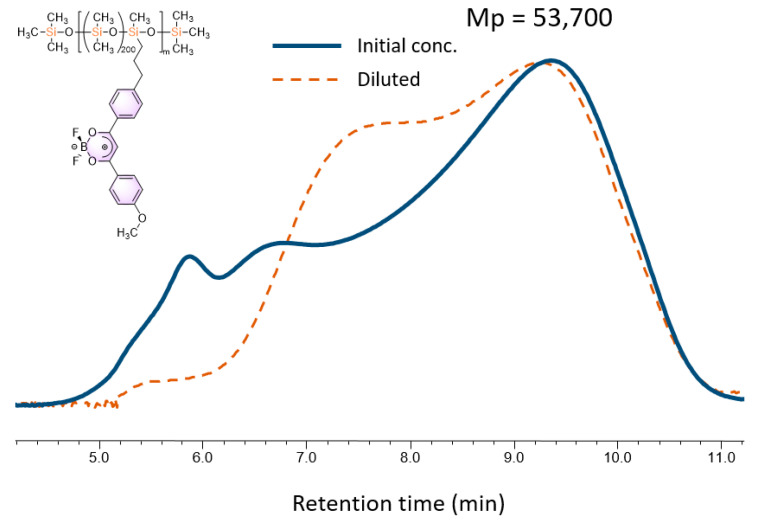
GPC curves of polymer **3 b** obtained in THF using column Phenogel 10^5^ Å.

GPC analysis of prepared polymers, performed in THF at 40 °C using column Phenogel 10^5^ Å (Figure 2 and Figure 3), showed that at these conditions the studied polymers had the following molecular characteristics: M_n_ = 49,400 Da, M_w_ = 453,100 Da, PDI = 9.2 for **3 a** and M_n_ = 64,500 Da, M_w_ = 244,100 Da, PDI = 3.8 for **3 b**. A broad and multimodal molecular weight distribution can be explained by the strong interchromophoric interaction between DBMBF_2_ fragments, because it is well known that DBMBF_2_ derivatives tend to aggregate and form H-dimers in solution as well as in the solid state [61]. An argument in favor of this assumption is the fact that the dilution of polymers **3 a,b** in solutions six times gives changes in molecular weight distribution, as can be seen from the GPC curves presented in Figure 2 and Figure 3. The more visible changes in the case of polymer **3 b** are related to a much lower content of DBMBF_2_ fragments in polysiloxane.

**Figure 4:**
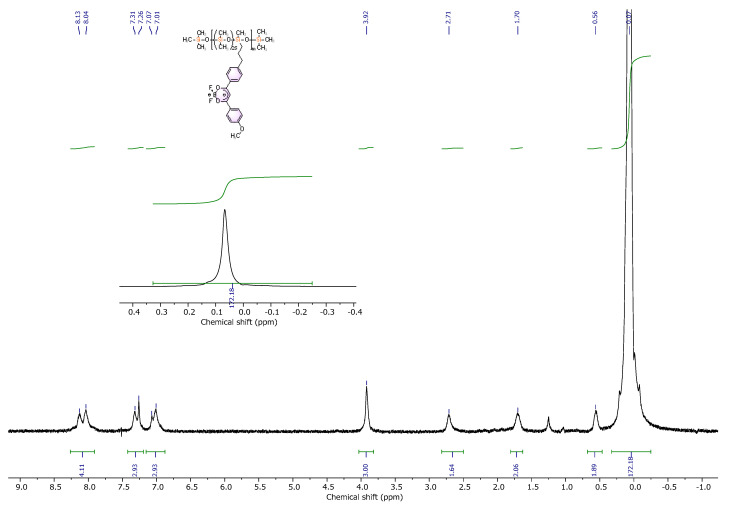
^1^H NMR spectrum of polymer **3 a** in CDCl_3_.

**Figure 5:**
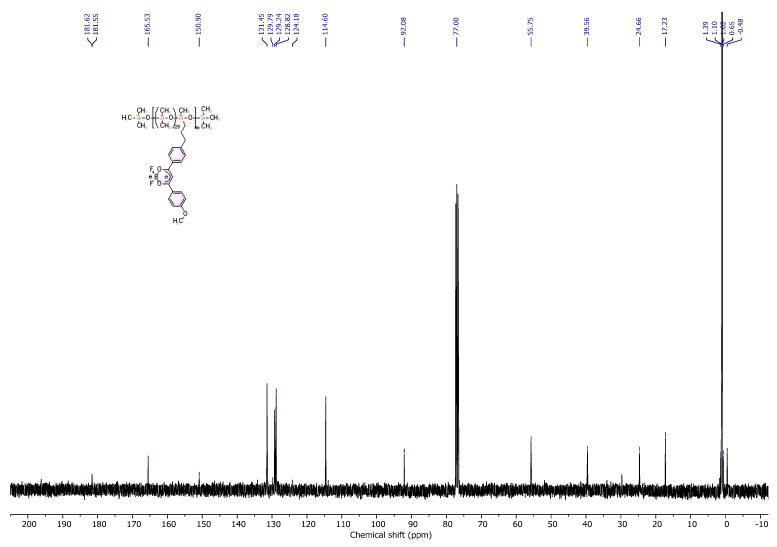
^13^C NMR spectrum of polymer **3 a** in CDCl_3_.

**Figure 6:**
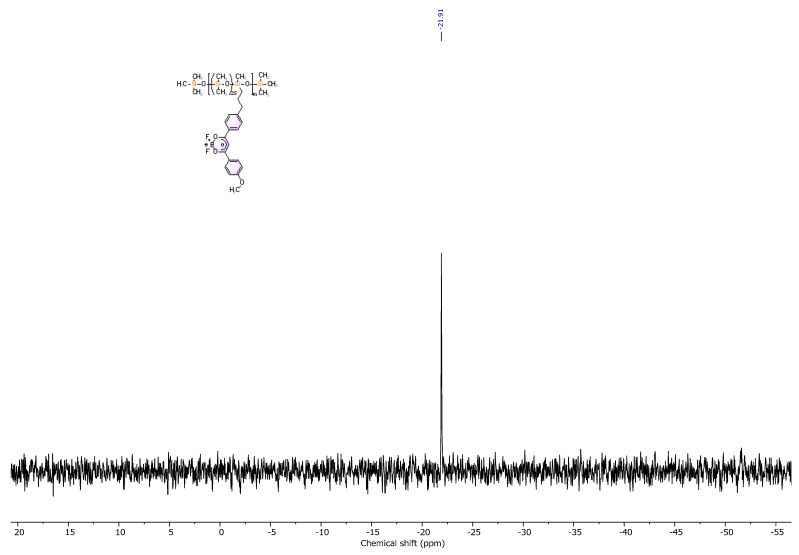
^29^Si NMR spectrum of polymer **3 a** in CDCl_3_.

**Figure 7:**
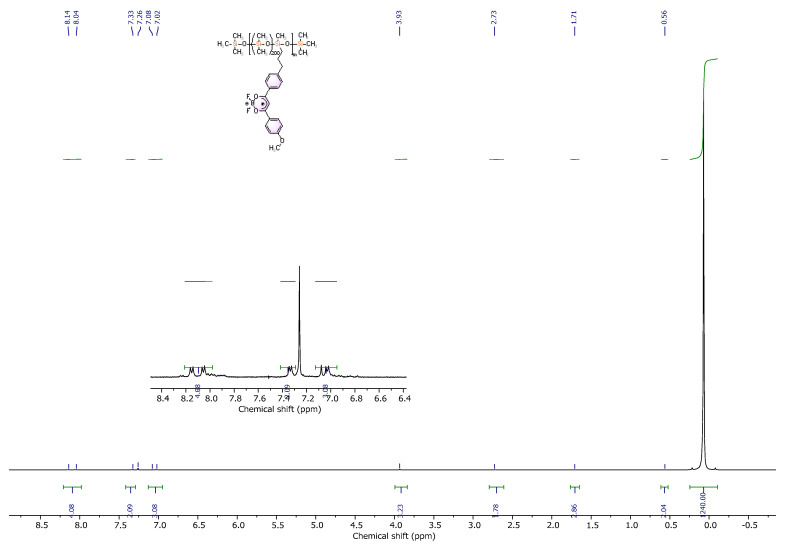
^1^H NMR spectrum of polymer **3 b** in CDCl_3_.

**Figure 8:**
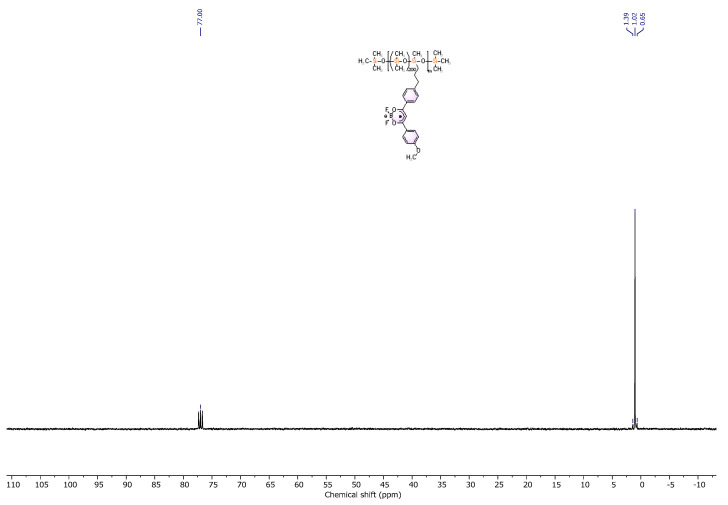
^13^C NMR spectrum of polymer **3 b** in CDCl_3_.

**Figure 9:**
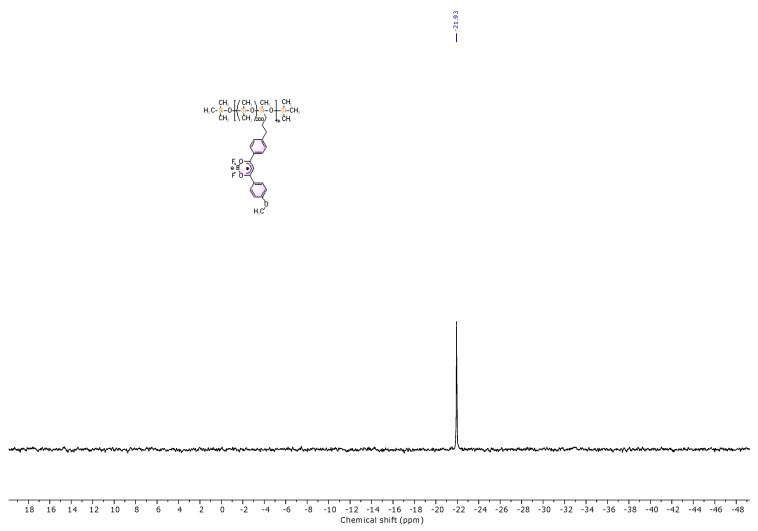
^29^Si NMR spectrum of polymer **3 b** in CDCl_3_.

The completeness of the reaction was confirmed by NMR method (Figure 4, Figure 5, Figure 6, Figure 7, Figure 8 and Figure 9), which showed the disappearance of signals assigned to Si-H in the ^1^H NMR (Figure 4 and Figure 7) and FTIR spectra (Figure 1), as well as the appearance of new signals assigned to DBMBF_2_ fragments. In FTIR spectra, new bands appear at 1500–1600 cm^−1^ which relate to C=O stretching vibrations of DBMBF_2_ fragments (Figure 1). ^1^H NMR spectra of polymers **3 a,b** contain new signals that correspond to protons of propyl fragments (tree multiplets at 0.56, 1.70 and 2.71 ppm), aromatic protons of dibenzoylmethanato fragment (7.01–8.13 ppm) and CH proton of dicarbonyl group COCHCO (singlet at 7.06) (Figure 4 and Figure 7). ^13^C NMR spectrum of polymer **3 a** contains all carbon signals corresponding to the structure of polymer (Figure 5). At the same time, in ^13^C NMR spectrum of polymer **3 b**, only carbon signals of -(CH_3_)_2_Si-O- segment that is related to a small concentration of DBMBF_2_ fragments in the polymer can be seen (Figure 8). ^29^Si NMR spectra of polymers **3 a,b** contain one signal at 21.9 ppm that corresponds to O-(CH_3_)_2_Si-O- segment (Figure 6 and Figure 9). The absence of other signals in the spectrum, for example from silsesquioxane fragments, tells us that the hydrosilylation reaction occurs regioselectively and gives only the β-product.

### 3.2. Characterization

**Figure 10:**
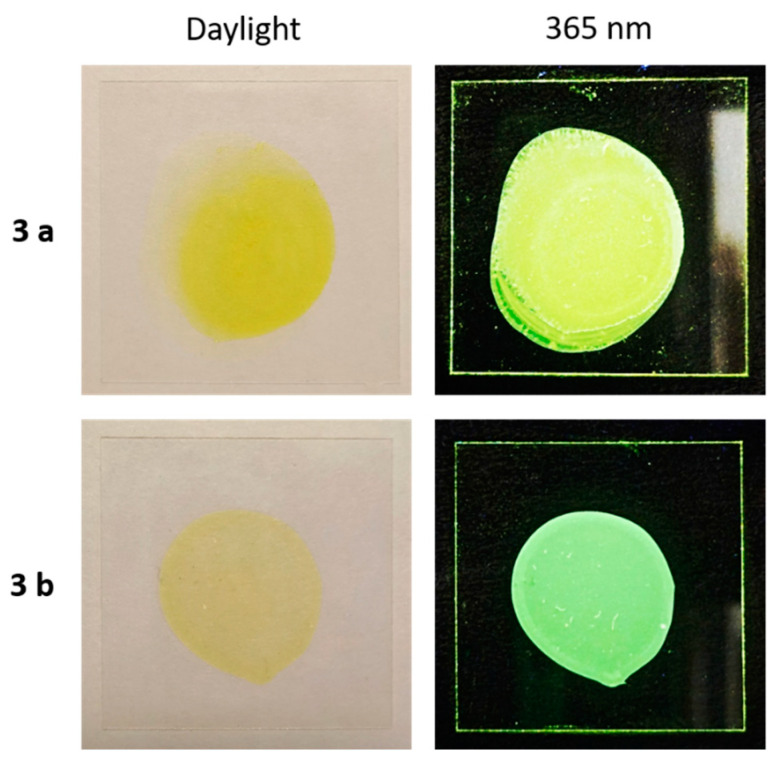
Photographs of polymers **3 a,b** in daylight and under excitation with light 365 nm.

**Figure 11:**
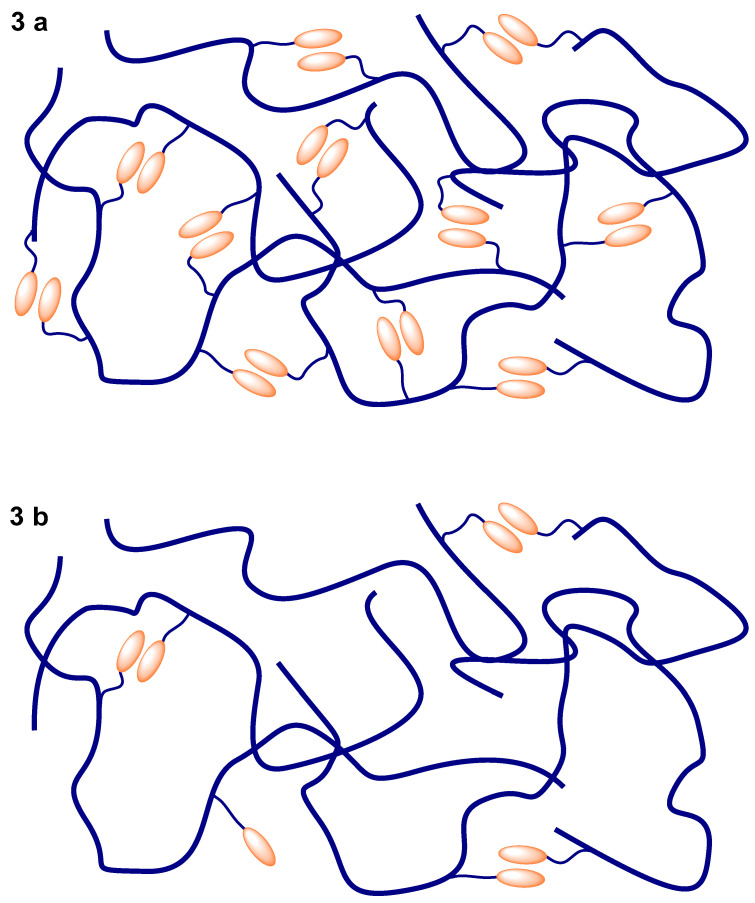
Schematic representation of polymers **3 a,b**.

Precursor polymers **1 a,b** are colorless viscous oils, while synthesized MeO-DBMBF_2_-grafted polymers **3 a,b** are yellow elastic solids which have intensive yellow (**3 a**) and yellow-green (**3 b**) fluorescence under excitation with light 365 nm (Figure 10). As mentioned above, DBMBF_2_ derivatives tend to aggregate and form H-dimers due to a high dipole moment of the DBMBF_2_ moiety (about 6,7 D) [64]. This explains the presence of strong intermolecular interactions leading to the appearance of elastomeric properties in polymers **3 a,b**. The structures of polymers **3 a,b**, where MeO-DBMBF_2_ content is almost an order of magnitude different, could be presented schematically as shown in Figure 11.

### 3.3. TGA, DSC and TMA Analysis

**Figure 12:**
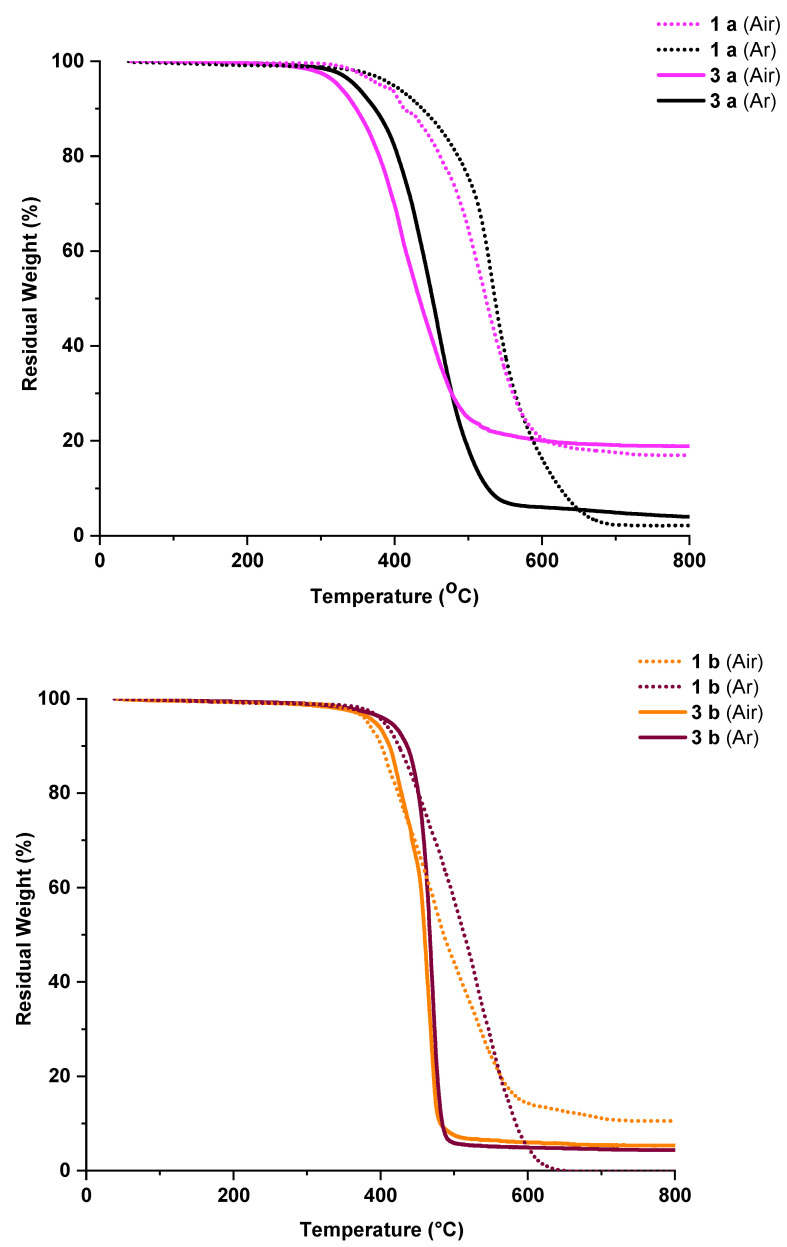
Thermogravimetric analysis (TGA) curves for compounds **1 a,b** and **3 a,b** in air and argon at the heating rate of 10 °C/min.

The thermal behavior of polymers **3 a,b** was investigated using thermal gravimetric analysis (TGA), differential scanning calorimetry (DSC) and thermomechanical analysis (TMA). The thermal stability of polymers **1 a,b** and **3 a,b** was estimated using the TGA method, both in air and in argon, in the range from 50 to 800 °C, and corresponding data are presented in Figure 12 and Table 1.

According to the TGA data, the thermal and thermo-oxidative stability of silylhydride-contained PDMS **1 a,b** are close to the reference data observed for PDMS (about 380 °C in air and 400 °C in argon) [65]. The inclusion of a small amount of DBMBF_2_ fragments into PDMS (case of **3 b**) leads to a small increase in thermal (T_d_^5%^ = 393 °C) as well as thermo-oxidative (T_d_^5%^ = 413 °C) stability, which may be due to the disappearance of Si-H groups tending to oxidation. In contrast, polymer **3 a**, which has a large amount of DBMBF_2_ fragments in the structure, is much less stable than precursor **1 b** in air as well as in argon atmosphere (T_d_^5%^ of **3 a** is 323 °C in air and 347 °C in argon).

**Figure 13:**
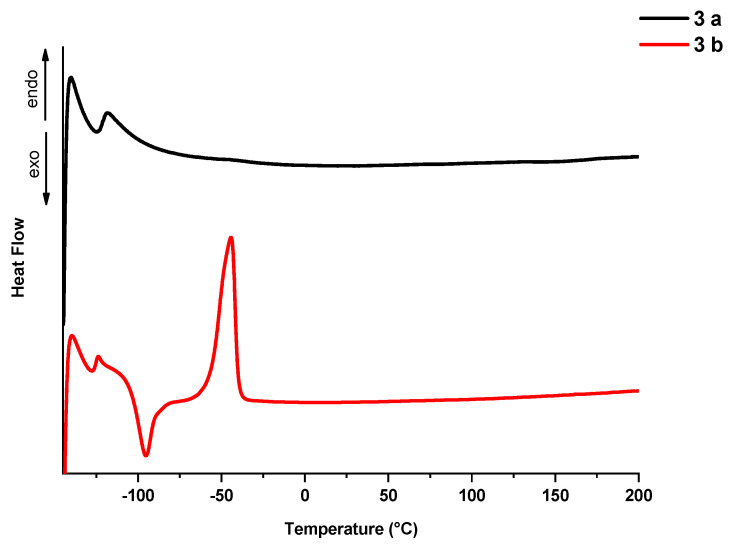
DSC curves for polymers **3 a,b** at a heating rate of 10 °C/min.

For polymers **3 a,b** DSC analysis was carried out. DSC traces for studied polymers are presented in Figure 13 and the glass transition temperatures as well as the observed thermal effects are summarized in Table 1. As can be seen from Figure 13, incorporation of a small amount of DBMBF_2_ fragments in PDMS (polymer **3 b**) leads to a partial inhibition of crystallization in comparison with PDMS [66] which is expressed as a monomodal melting peak at −44 °C. At the same time, in the case of polymer **3 a** the crystallization ability of PDMS is completely suppressed.

**Figure 14:**
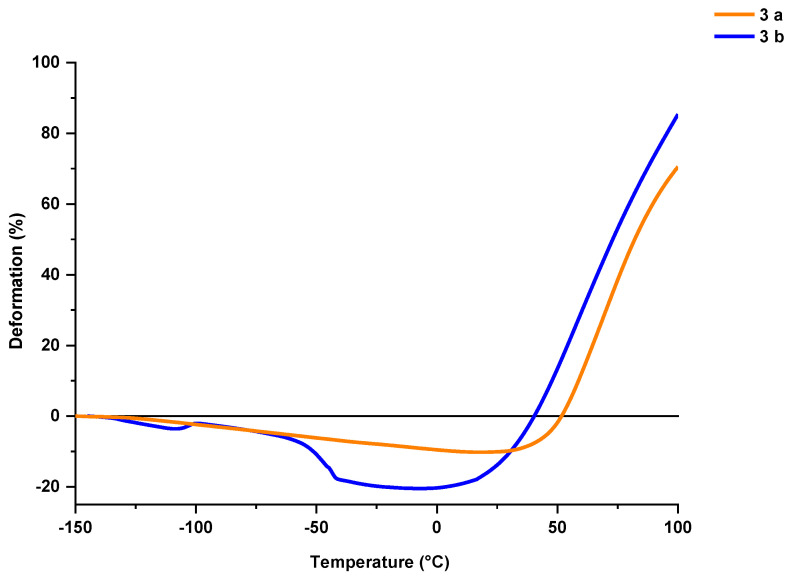
TMA curves for polymers **3 a,b** at a heating rate of 5 °C/min in dilatometric mode with a constant load of 0.01 N.

TMA analysis of polymers **3 a,b** showed that in TMA curves of all samples, a deformation which was associated with the transition from the glassy state to the highly elastic state and then to the viscous flow state is observed (Figure 14). The start of the transition to a viscous flow state for polymer **3 a** was observed at a higher temperature (about 40 °C) than in the case of polymer **3 b** (about 20 °C).

### 3.4. Rheological Properties

**Figure 15:**
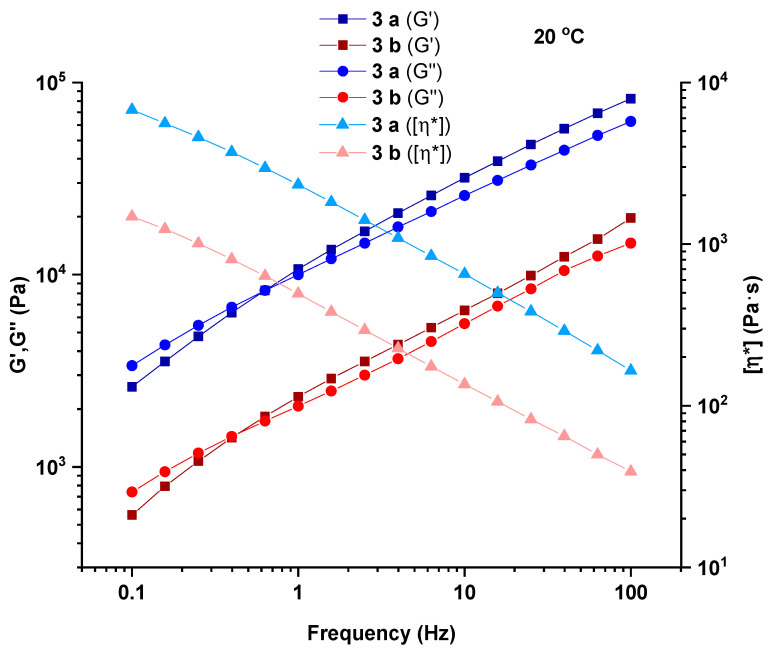
Frequency dependences of G′, G″ and [η*] for polymers **3 a,b** at 20 °C.

The viscoelastic characteristics of polymers **3 a,b** (storage modulus (G′), loss modulus (G″), complex viscosity ([η*])) were determined in the sinusoidal oscillating vibrations mode. Figure 15 shows frequency dependences of G′, G″ and [η*] for polymers **3 a,b** at 20 °C. From the frequency dependences presented in Figure 15, it can be seen that polymers **3 a,b** at low frequencies at room temperature are elastic-viscous liquids (G′ < G″). Moreover, G′, G″ and [η*] for **3 a** exceed the similar values for **3 b**. Increasing the frequency leads to inversion (crossover) of the dependences of G′ and G″, which indicates the transition of polymers from viscous to elastomeric behavior characteristics and the beginning of the formation of a physical network (according to Graessley) [67].

**Figure 16:**
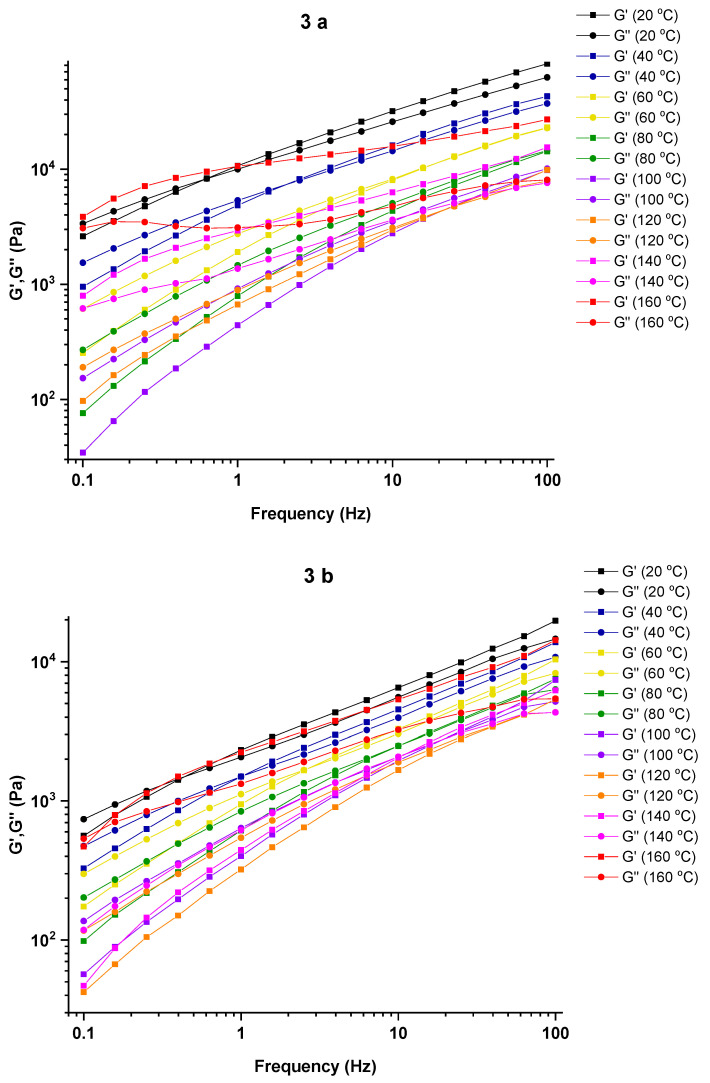
Frequency dependences of G′, and G″ for polymers **3 a** (**top**) and **3 b** (**bottom**) at different temperatures.

**Figure 17:**
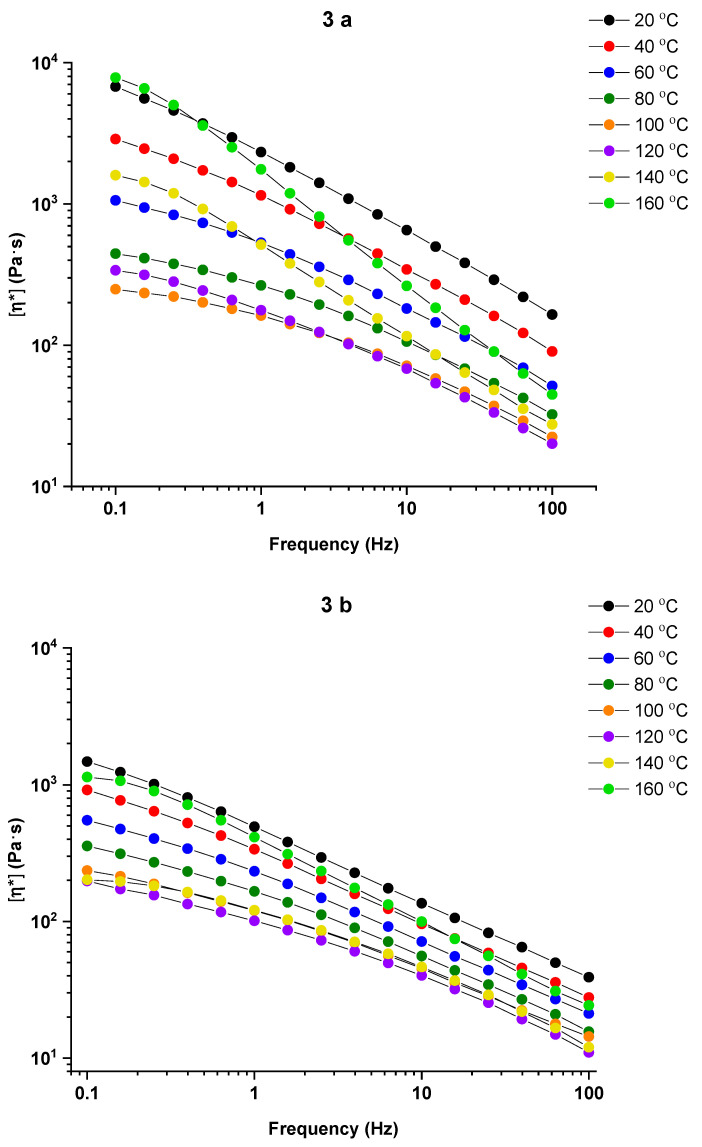
Frequency dependences of [η*] for polymers **3 a** (**top**) and **3 b** (**bottom**) at different temperatures.

**Figure 18:**
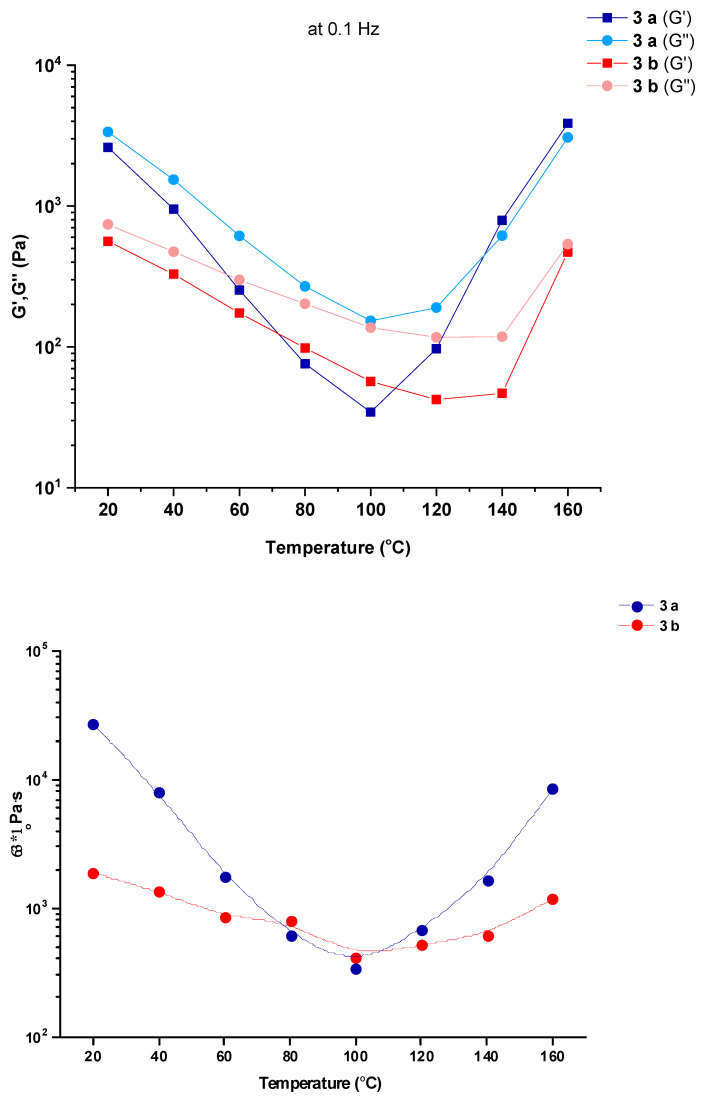
Frequency dependences of G′,G″ (**top**) and [η*]_0_ (**bottom**) for polymers **3 a**,**b** at different temperatures.

**Figure 19:**
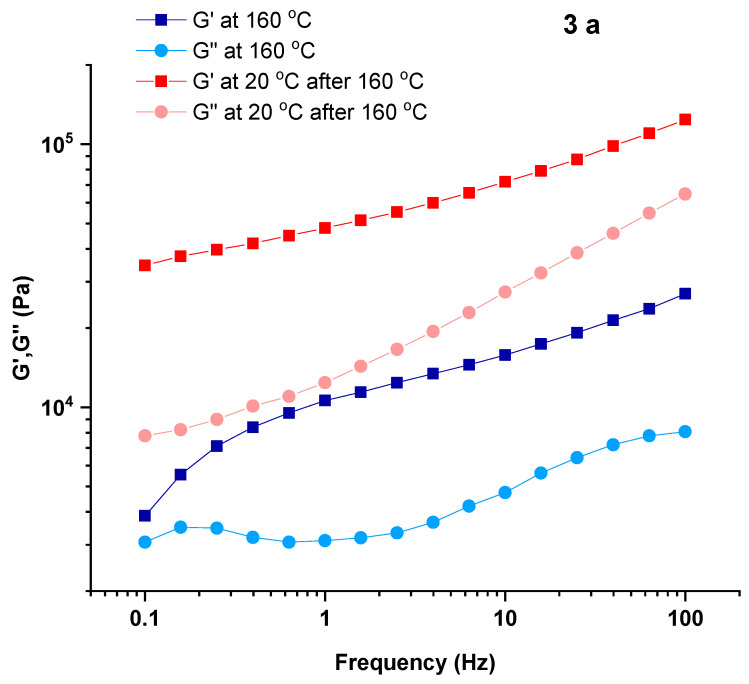

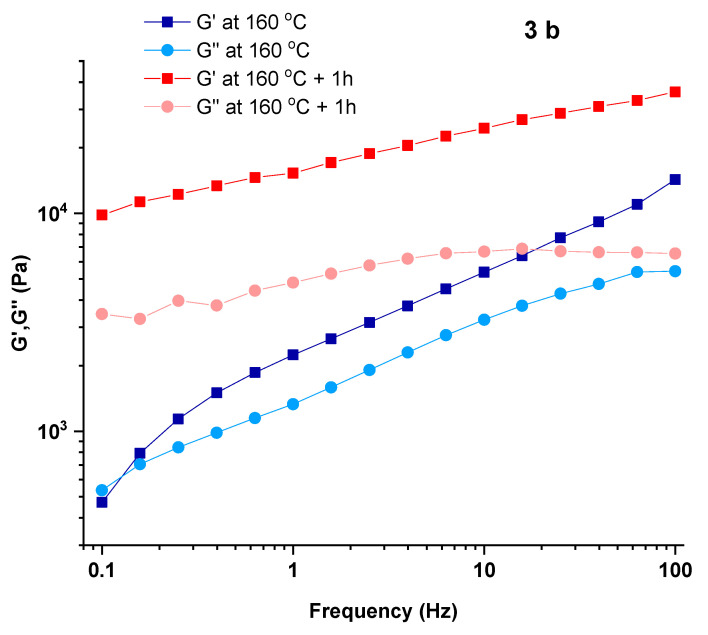
Frequency dependences of G′, and G″ for polymers **3 a** (**top**) at 160 °C and after cooling to 20 °C, and for polymer **3 b** (**bottom**) at 160 °C and after heating at 160 °C for 1h.

As the temperature rises to 100 °C there is a decrease in G′, G″ and [η*] in the entire frequency range for both polymers (Figure 16 and Figure 17). At the same time, the crossover position is shifted to higher frequencies. When the temperature rises above 120 °C, an increase in G′, G″ and [η*] is observed, which continues up to 160 °C. At this temperature, there is a qualitative change in the frequency dependence of G′ and G″ (Figure 18, top). On the frequency dependence of G′, a pronounced plateau of high elasticity appears, indicating the formation of a fairly stable network structure. This is especially noticeable for polymer **3 a** with the high content of DBMBF_2_ fragments. Figure 18 (bottom) shows the dependences of [η*]_0_ on temperature, where [η*]_0_ is the zero-shear-rate complex viscosity calculated by the Carreau–Yasuda model [68]. For polymers **3 a,b** a viscosity minimum is observed at 100 °C. It can be assumed that as a result of an increase in the mobility of macromolecules, the transition from intramolecular interactions to intermolecular ones becomes possible, which leads to the formation of a strong network structure. The spatial grid formed at 160 °C is quite stable, and after an additional heating of the polymer in the measuring unit of the viscometer for an hour G′ increases (Figure 19). After the polymers are cooled from 160 °C to 20 °C, their elastic modulus increases (Figure 19), which may indicate the formation of a larger number of network nodes.

**Figure 20:**
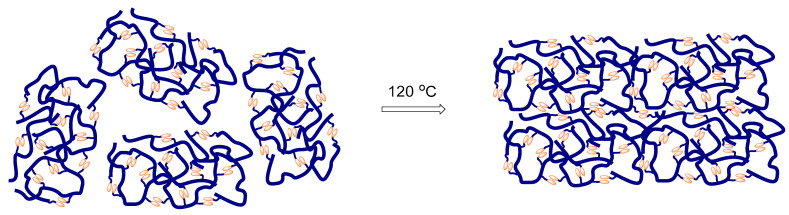
Schematic representation of the physical network formation in polymers **3 a,b** after heating to 120 °C.

The heating of polymers at temperatures above 120 °C is accompanied by an increase in [η*], G′ and G″ due to the formation of a spatial network of physical bonds at high temperatures. The formation of such networks during the heating of polydimethylsiloxanes containing carboxyl, amide, and ester groups, which is prone to the formation of specific interactions, was observed earlier [69,70,71]. In our case the strong interchromophoric interaction between DBMBF_2_ fragments causes the formation of a stable physical network. The observed formation of the spatial network structure with increasing temperature is associated with a change in the type of aggregation of DBMBF_2_ groups, leading to a change in the initial conformation of siloxane chains, which is determined by the conditions of synthesis and isolation of modified PDMS (Figure 20). After the synthesis and subsequent reprecipitation in a thermodynamically poor solvent (THF), siloxane macromolecules acquire a more twisted conformation than they have in a good solvent. This conformation is stabilized by the intramolecular pair interactions of DBMBF_2_ groups (Figure 11). With an increase in temperature the dissociation of intramolecular physical bonds between functional groups becomes possible, due to the specifics of the siloxane chain, and a statistical reorganization of intramolecular bonds into intermolecular ones occurs [72,73]. The resulting network of physical bonds is quite stable and even increases the number of elastically active chains at 160 °C, as evidenced by the increase in G’ after additional heating. The possibility of its full dissolution in boiling chloroform, during a gel fraction analysis, testifies to the reversibility of the network.

### 3.5. Optical Properties

**Figure 21:**
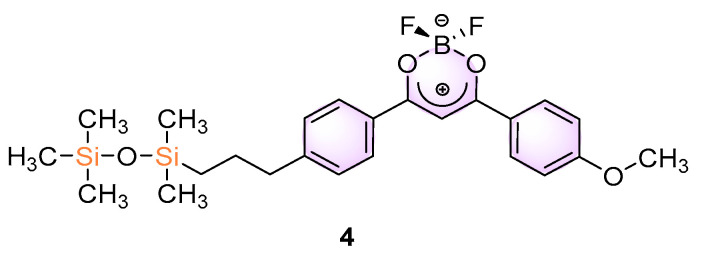
Model compound **4**.

The optical properties of the synthesized compounds were studied in solutions in various solvents at room temperature, as well as in the solid state, and are summarized in Table 2. DBMBF_2_ derivative **4**, described earlier by us [63], was used as a monomeric model compound for the comparison with studied DBMBF_2_-grafted polymers **3 a,b** (Figure 21).

**Figure 22:**
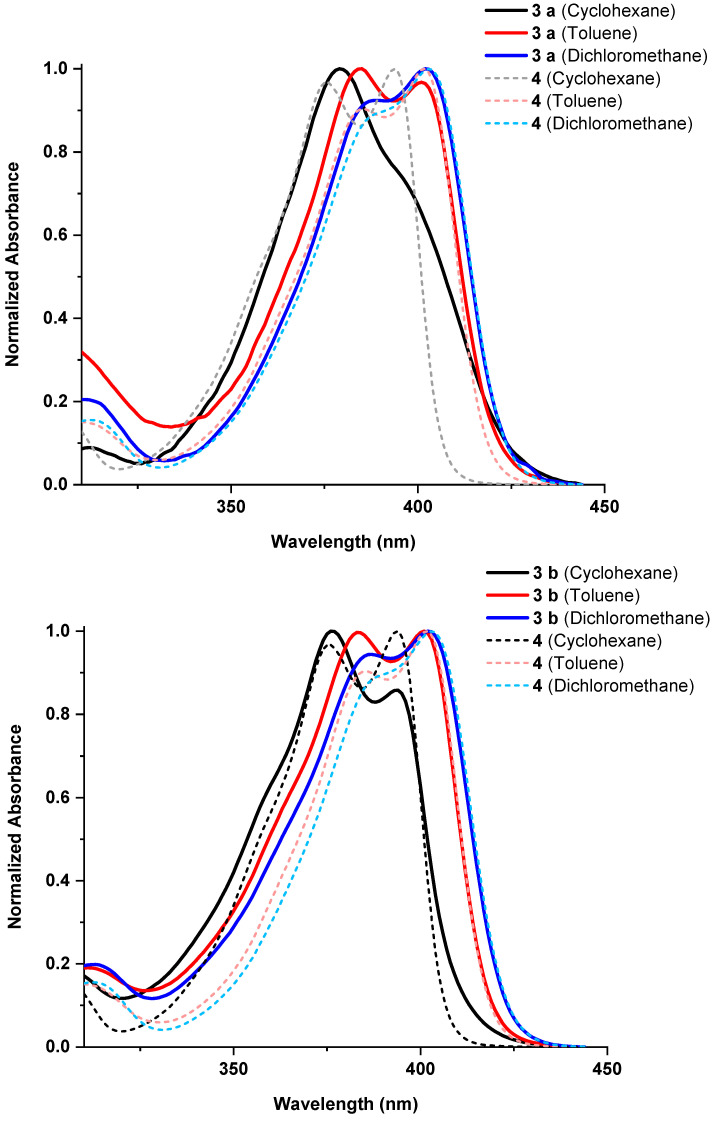
Normalized UV-visible absorption spectra of polymers **3 a,b** in various solvents (RT, OD < 1).

The normalized UV-visible absorption spectra of DBMBF_2_-grafted polymers **3 a,b** and model compound **4** in cyclohexane, toluene and dichloromethane at room temperature are shown in Figure 22. The absorption spectra of model compound **4** in toluene and dichloromethane consist of strong bands similar in structure, attributed to π-π^*^ transitions. In toluene and dichloromethane, the bands first peak at about 402 nm, with less intense second peaks at about 385 nm and shoulders at about 365 nm, relating to the 0–0, 0–1, 0–2 vibronic transitions, respectively. 

On the contrary, in cyclohexane, the absorption spectrum of **4** is slightly hypsochromically shifted with peaks at approximately 394 nm (maximum), 376 nm and 355 nm (shoulder). The absorption spectra of polymers **3 a,b** have relative intensities of vibronic transitions different from model compound **4**. It is noteworthy that in the case of nonpolar solvents, such as cyclohexane, the intensity of the 0–0 transition drops significantly compared with the 0–1 transition. This behavior indicates the existence of intramolecular H-dimers in the ground state and has been described in detail in our previous work [62]. As shown in Figure 22, in the case of polymer **3 a** with a higher concentration of grafted DBMBF_2_ fragments, the content of H-dimers is much higher than in the case of polymer **3 b**. Additionally, the content of H-dimers strongly depends on the solvent polarity and is highest in less polar solvents, such as cyclohexane.

**Figure 23:**
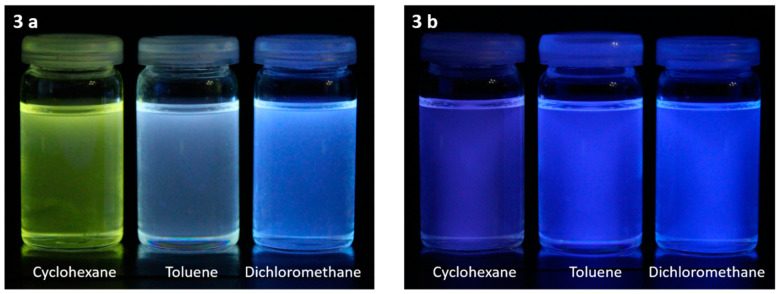
Photographs of solutions of polymers **3 a,b** in cyclohexane, toluene and dichloromethane under UV light with a wavelength of 365 nm.

**Figure 24:**
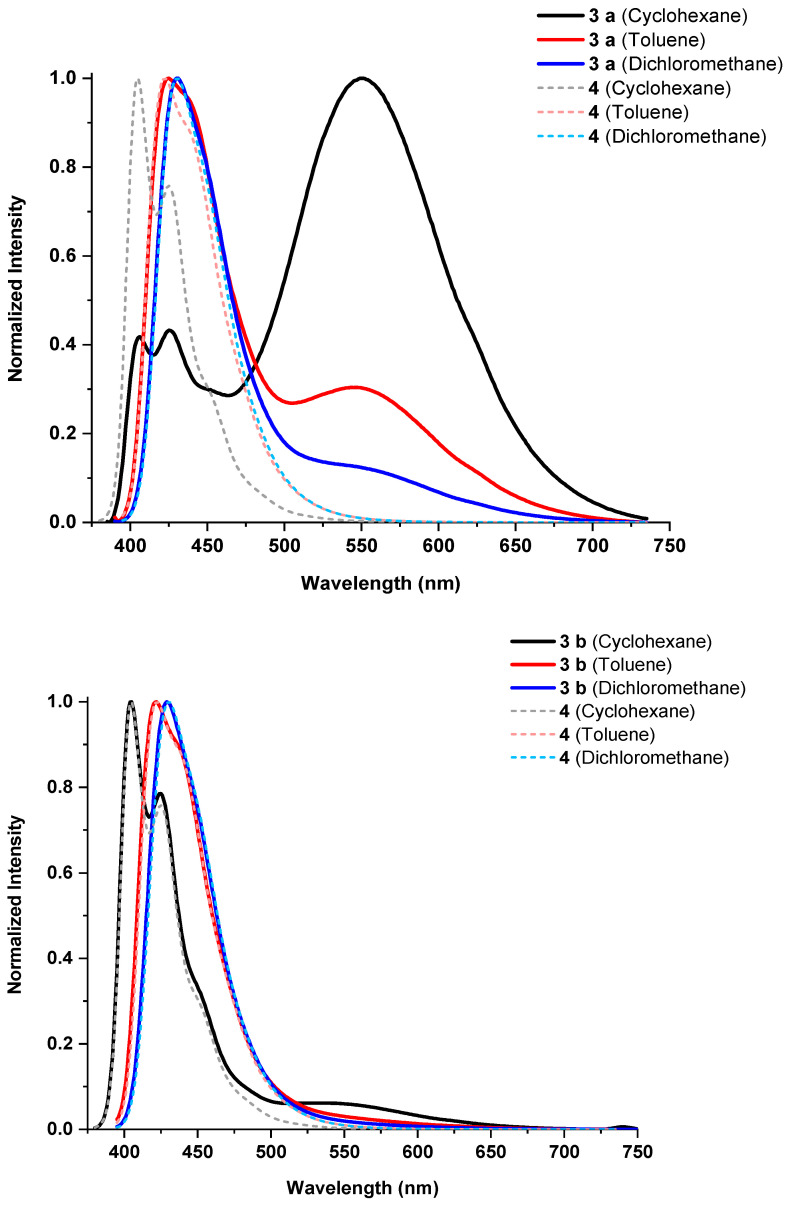
Normalized emission spectra of polymers **3 a,b** in various solvents (RT, OD < 0.1). Excitation at 375 nm.

The steady-state emission spectra of studied polymers **3 a,b** and model compound **4** were measured in cyclohexane, toluene and dichloromethane (c = 1 × 10^−6^ M) at room temperature. Figure 23 shows photographs of the studied solutions of polymers **3 a,b** under UV light with a wavelength of 365 nm. Fluorescence spectra of polymers **3 a,b** and model compound **4** are presented in Figure 24 and their optical properties are summarized in Table 2.

As can be seen in Figure 24, the fluorescence spectra of the model compound **4** in cyclohexane consist of two resolved peaks at approximately 405 and 425 nm and a shoulder at approximately 450 nm. In toluene and dichloromethane, the spectra are red-shifted with maxima at 422 (in toluene) and 430 (in dichloromethane) nm. The fluorescence quantum yield is in the range of 0.82–0.87 for the studied solvents. In the case of polymers **3 a,b**, the fluorescence spectra contain additional wide unstructured low-energy bands with maxima at about 550 nm. Such behavior has been observed earlier in similar siloxane-containing systems [61,62,63] and is related to the excimer-like fluorescence. Polymer **3 a**, which contained more frequently grafted DBMBF_2_ fragments (each 25th unit), tended to form many more excimers than polymer **3 b** with a sparse distribution of DBMBF_2_ fragments (each 200th unit), as clearly shown by the comparison of I_ex_/I_m_ ratio (Table 2). The solvent polarity has a significant effect on the excimer contribution to the overall emission spectrum. The fluorescence spectra with the largest concentration of the excimer-like emissions were observed in the case of cyclohexane. Figure 23 shows the visual differences in color of the emissions of polymers **3 a,b** in various solvents under UV light with a wavelength of 365 nm. The fluorescence quantum yield of polymers **3 a,b** drops significantly in comparison with model compound **4** (Table 2); this is obviously related to the high nonradiative constant of the formed excimers.

**Figure 25:**
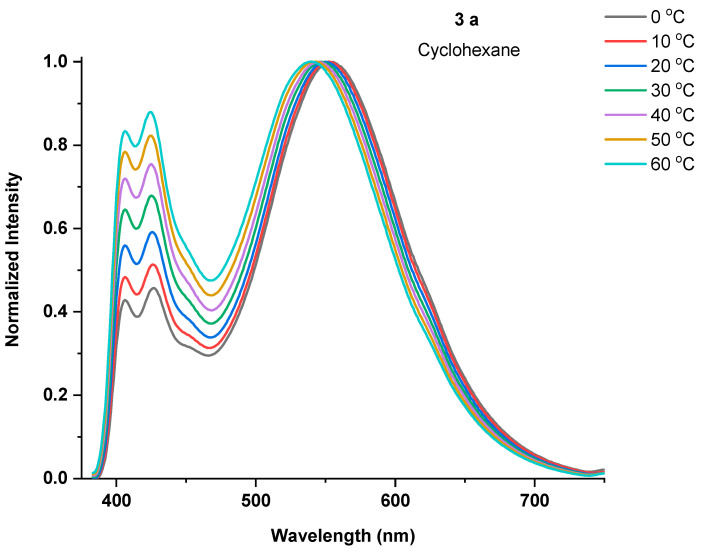

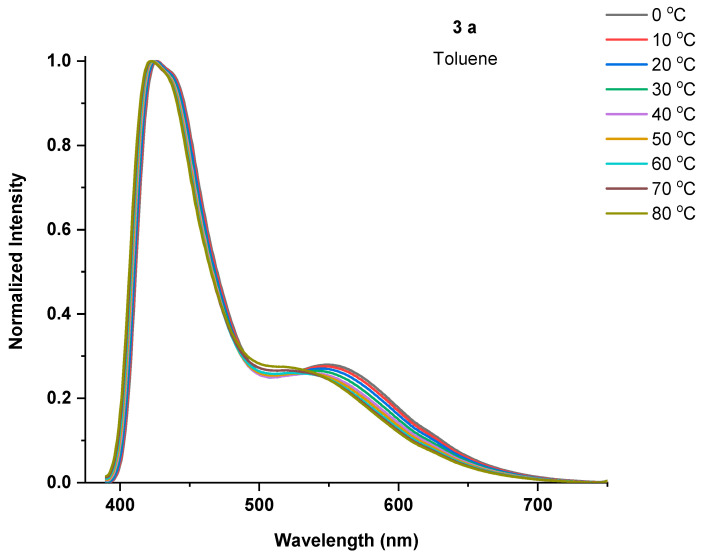
Normalized emission spectra of polymer **3 a** in cyclohexane (**top**) and toluene (**bottom**) at different temperatures.

**Figure 26:**
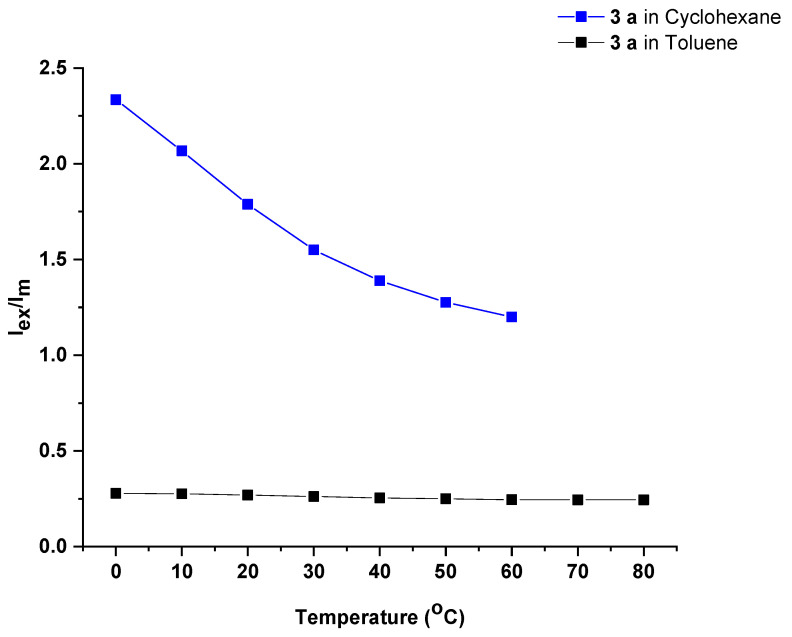
The I_ex_/I_m_ ratio dependence on the temperature in cyclohexane and toluene solutions of polymer **3 a** at different temperatures.

The influence of temperature on the fluorescence spectra was investigated and the results are presented in Figure 25. For this purpose, the fluorescence spectra for the solutions of polymer **3 a** in cyclohexane and toluene were measured at various temperatures from 0 to 60 °C for cyclohexane and from 0 to 80 °C for toluene. As shown in Figure 25, polymer **3 a** in cyclohexane shows a significant decrease in excimer fraction with an increase in temperature. The hypsochromic shift of the maximum of the excimer emission was also observed with the temperature increase. In contrast, in toluene solution for polymer **3 a**, only hypsochromic shift of the maximum of the excimer fluorescence and a barely noticeable decrease in the excimer emission with the temperature increase were observed. The corresponding I_ex_/I_m_ ratios at different temperatures were calculated using the aforementioned equation and are summarized in Table 3 and Figure 26.

**Figure 27:**
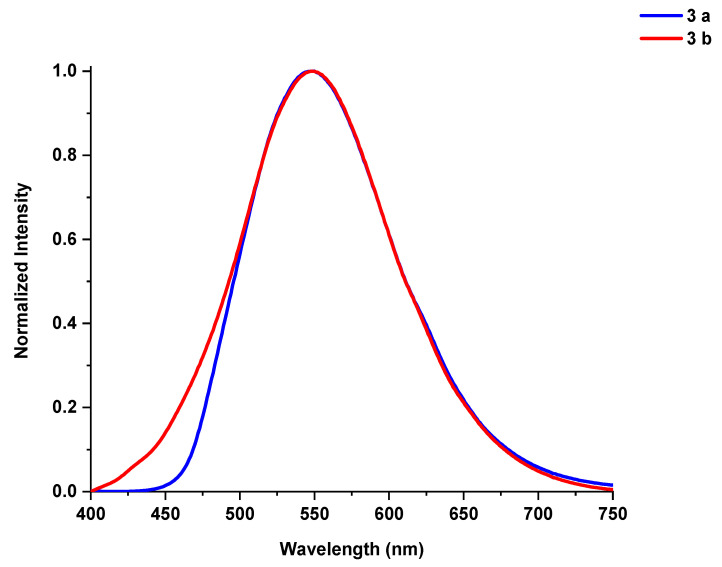
Normalized emission spectra of polymers **3 a,b** in the solid state. Excitation at 375 nm.

**Figure 28:**
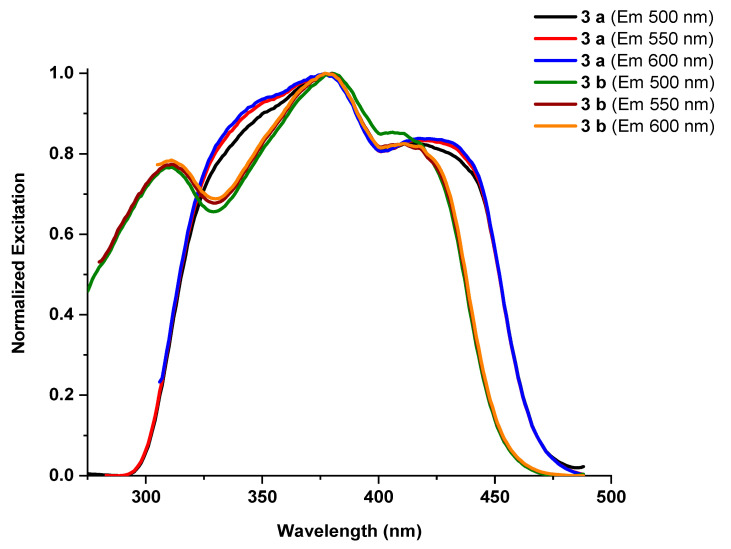
Normalized fluorescence excitation spectra of polymers **3 a,b** in the solid state.

The emissive properties of polymers **3 a,b** were also examined in the solid state. Polymer **3 a** demonstrated yellow (λ_em_ = 550 nm), and **3 b** greenish yellow (λ_em_ = 550 nm) fluorescence when excited by light, with a wavelength of 365 nm (Figure 10 and Figure 27). Figure 28 shows the normalized fluorescence excitation spectra of polymers **3 a,b** in the solid state. As can be seen from the excitation spectra, in the case of polymer **3 a**, which contains about tenfold more DBMBF_2_ fragments than polymer **3 b**, a large number of aggregates are formed, as evidenced by the broadened band in the long-wavelength region of the spectrum.

**Figure 29:**
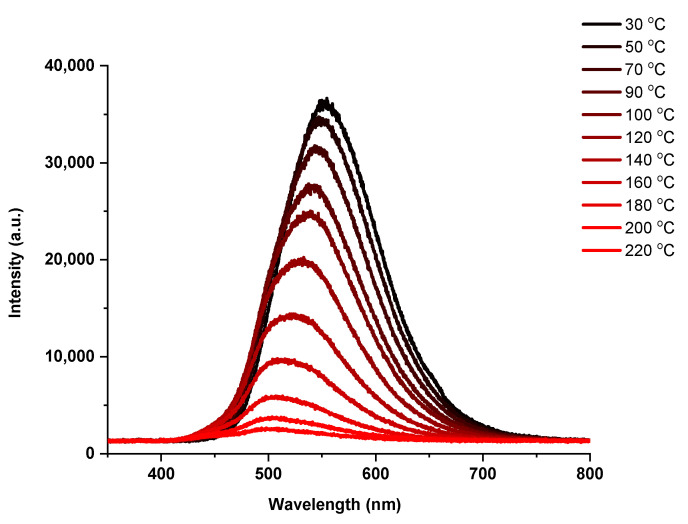
Emission spectra of polymer **3 a** in the solid state at different temperatures.

The fluorescent properties of polymer **3 a** in the solid state at different temperatures were studied and the results are presented in Figure 29. As shown, during the transition from 30 to 220 °C the intensity of fluorescence drops drastically and a shift of maximum fluorescence to the short-wavelength region is observed. Therefore, this DBMBF_2_-grafted polymer can be used as a fluorescent temperature sensor.

**Figure 30:**
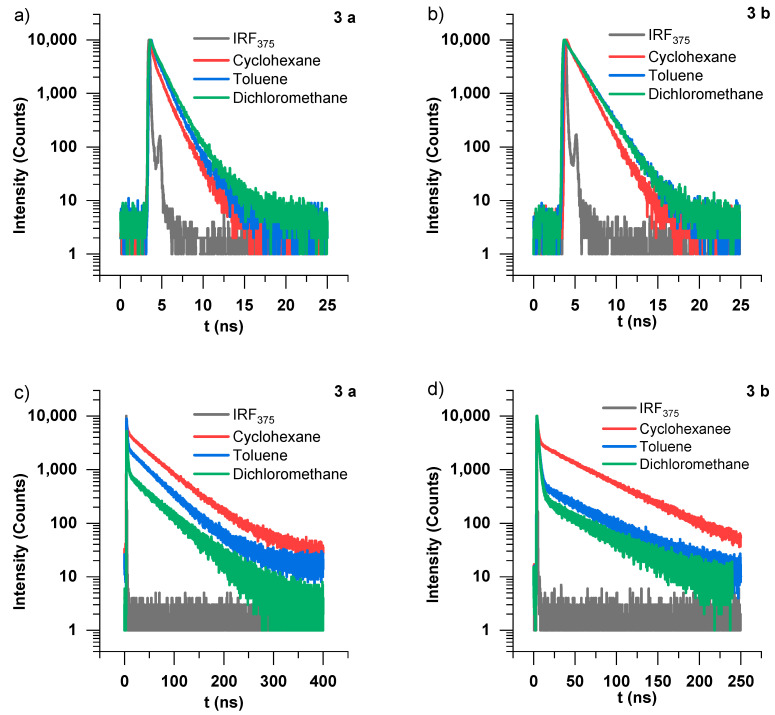
Fluorescence decay curves for polymers **3 a** (**a**) and **3 b** (**b**) at 436 nm and **3 a** (**c**) and **3 b** (**d**) at 540 nm in cyclohexane, toluene and dichloromethane compared with instrument response function (IRF) (λ_ex_ = 375 nm).

The fluorescence decays obtained for polymers **3 a,b** at 436 and 540 nm are shown in Figure 30. The decays are nonexponential. The two-exponential model is sufficient to fit the decays measured for polymer **3 b** in all cases, although only one lifetime obtained at two wavelengths matches. In the case of polymer **3 a** in all solvents, three exponential terms are required to fit fluorescence decays at 436 nm. Only two terms are needed to satisfactorily describe the kinetics at 540 nm in cyclohexane and toluene, and three terms are required for solution in dichloromethane. The fitting results are presented in Table 4. The fluorescence kinetics at 436 nm become longer with the increase of solvent polarity in the series of cyclohexane, toluene, and dichloromethane. The fluorescence decays at 540 nm consist of initial fast kinetics and a subsequent slower decay with lifetime around 50 ns, which, according to the literature, corresponds to an excimer fluorescence. The contributions of fast kinetics increase with increasing solvent polarity. The obtained kinetics resemble the decays obtained for the DBMBF_2_ connected with diphenylsiloxane linkers described previously by us [62], although the contribution of fast kinetics at 540 nm is much higher. The same model discussed for the dyads may be proposed for polymer **3 b**, but the results obtained for polymer **3 a** and the third exponential term observed at 540 nm for **3 b** in dichloromethane indicate that the observed fluorescence kinetics are more complex in nature.

**Figure 31:**
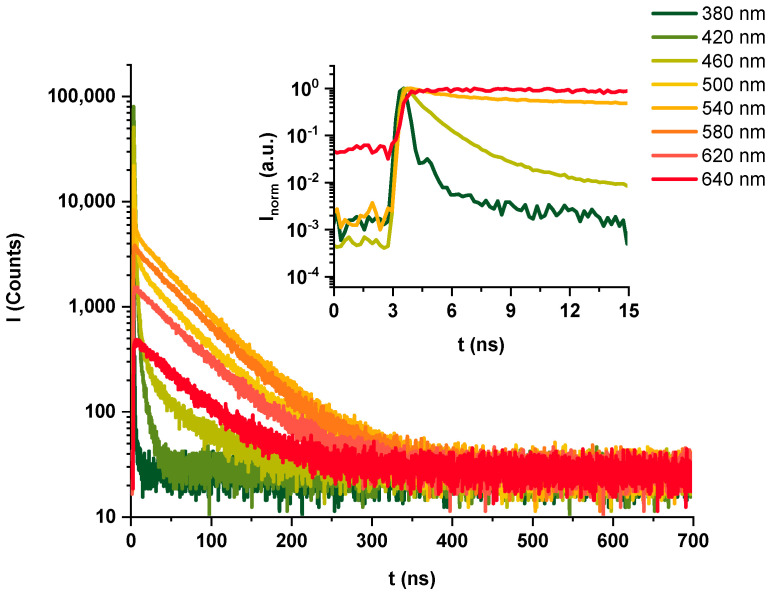
Fluorescence decay curves for polymer **3 a** at a series of wavelengths in the range of 380–660 nm in cyclohexane (λ_ex_ = 375 nm). In the inset, the comparison of normalized decays obtained at 380, 460, 540 and 660 nm is shown.

The fluorescence decays at wavelengths in the range of 380–700 nm were measured for further investigation. The fluorescence kinetics obtained for polymer **3 a** in cyclohexane at a series of wavelengths are presented in Figure 31. The comparison of normalized kinetics obtained at four wavelengths is presented in the inset. The rise process can be seen by comparing the kinetic obtained at 640 nm with that obtained at 380 nm. The fluorescence spectra obtained at a given time from the decay curves in Figure 31 and normalized at maximum are shown in Figure S11 (SI). The rise of the new fluorescence band is observed in the range of 500–600 nm, whereas the initial fluorescence band in the range of 400–500 nm fades almost completely within 10 ns. 

The satisfactory global fitting of the obtained time-resolved spectra requires four exponential terms. The results of global fitting for all solvents are presented in Table 4. The intensities spectra corresponding to four exponential terms in comparison with the steady-state fluorescence spectra are shown in Figure S12 (SI) for polymer **3 a**. The main difference between polymers and dyads is the presence of the fourth exponential term which has the spectrum maximum at about 500 nm and shorter lifetime around 10 ns. It can be assumed that this spectrum corresponds to some complexes in the excited state with geometry different from the equilibrium geometry of excimers. The formation of such complexes is apparently the result of the steric hindrances of the fluorophore motion arising from the interaction with polymer chains.

The dependence of fluorescence kinetics on wavelength for the solid state samples of polymers **3 a,b** are presented in Figure S13 (SI). The time-resolved fluorescence spectra of **3 a** shown in Figure S14 (SI) demonstrate the gradual shift of the spectral band maximum to longer wavelengths over time. The global fitting results show that the obtained time-resolved fluorescence spectra can only be satisfactorily described by a five-exponential model (see Table S2 (SI) for lifetimes). The intensities maxima of the spectra corresponding to the exponential terms gradually shift to the longer wavelength with the increase of lifetime (Figure S15 (SI)). Thus, the observed behavior may be associated with the presence of a diversity of aggregates with various geometries in the solid state samples of polymers **3 a,b** and the degree of steric hindrance to the movement of the fluorophore.

**Figure 32:**
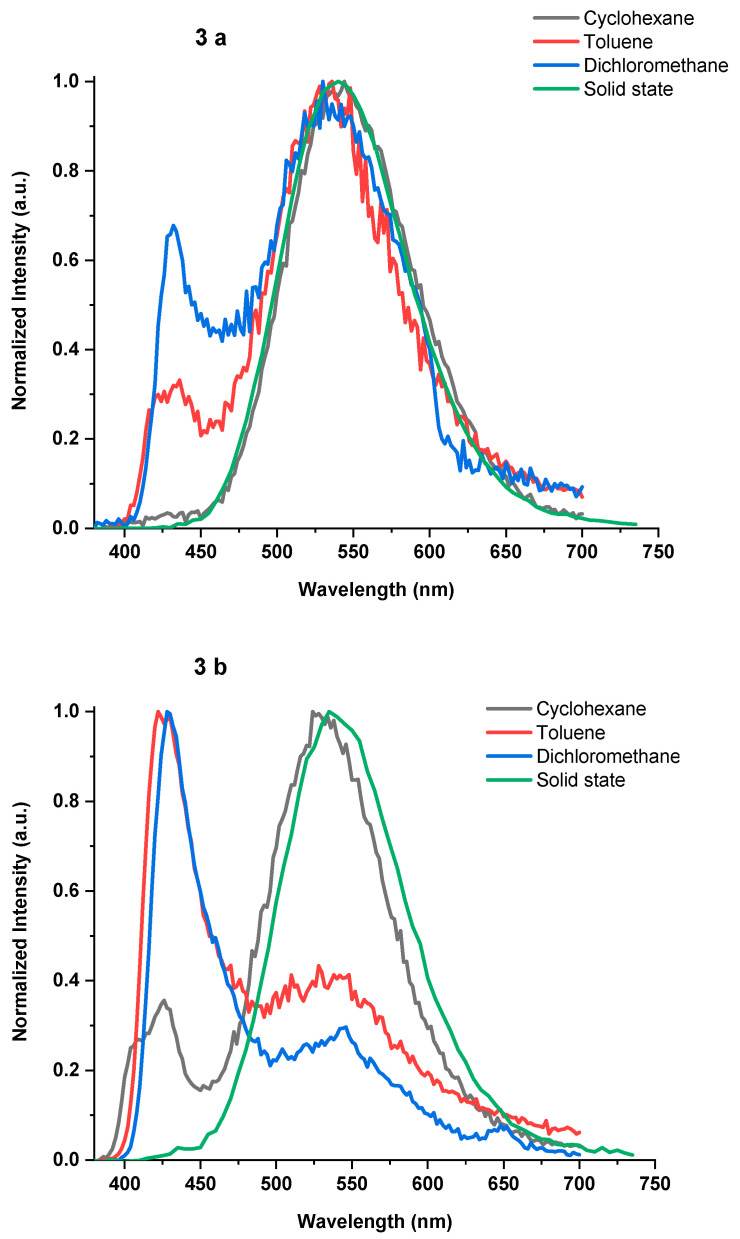
The delayed fluorescence spectra of polymers **3 a** (**top**) and **3 b** (**bottom**) in various solvents and solid state.

Delayed fluorescence was observed for polymers **3 a,b** in the solution and the solid state. The spectra of delayed fluorescence are shown in Figure 32. In the solid state, the delayed fluorescence of both samples has one nonstructured band with the maxima at 540 nm, which resembles the excimer fluorescence. As was reported previously, for this class of fluorophores the delayed fluorescence is thermally activated and arises due to aggregate formation [74,75]. Contrastingly, in the solution, delayed fluorescence spectra have two bands, the first with maximum at 430 nm and the second with maximum about 540 nm. The contribution of the band at 430 increases with increasing solvent polarity, apparently due to the increase of fluorophore mobility, and decreases with the rise of fluorophore concentration, due to the increase of the fluorophore aggregation degree. The delayed fluorescence is strongly quenched by oxygen in all cases. The kinetics of delayed fluorescence are nonexponential and can be fitted with two exponential terms (see Table 5). 

## 4. Conclusions

In summary, the method for the preparation of polydimethylsiloxanes with grafted dibenzoylmethanatoboron difluoride derivatives has been described. The structures of the prepared polymers were confirmed using NMR, IR spectroscopy and gel permeation chromatography methods. The thermal stability of the prepared polymers was investigated using thermal gravimetric analysis, which showed that the inclusion of a small amount of DBMBF_2_ fragments into PDMS leads to a small increase in thermal as well as thermo-oxidative stability. Contrastingly, the inclusion of a large amount of DBMBF_2_ fragments into PDMS leads to the reduction of thermal and thermo-oxidative stability of the polymer. The study of the thermal behavior of the polymers using differential scanning calorimetry analysis shows a full (for polymer **3 a**) or partial (for polymer **3 b**) suppression of crystallization in comparison with PDMS. TMA analysis indicates that the start of the transition to a viscous flow state for polymer **3 a** occurs at a higher temperature (about 40 °C) than in the case of polymer **3 b** (about 20 °C). The viscoelastic characteristics of the prepared polymers, such as storage modulus, loss modulus and complex viscosity, were determined in the sinusoidal oscillating vibrations mode. Polymers **3 a,b** at low frequencies at room temperature are elastic-viscous liquid (G′ < G″). Increasing the frequency leads to inversion (crossover) of dependences G′ and G″, which indicates the transition of polymers from viscous to elastomeric behavior characteristics and the beginning of the formation of a physical network. Heating of the polymers at temperatures above 120 °C is accompanied by an increase in [η*], G′ and G″ due to the formation of a spatial network of physical bonds at high temperatures. The study of optical properties was carried out using electron absorption, steady-state and time-resolved fluorescence spectroscopy. The absorption spectra of studied polymers indicate the existence of intramolecular H-dimers in the ground state. The polymers have a bright fluorescence in the solution and in the solid state. The emission spectra consist of the monomer and excimer emission bands. In the solid state, only excimer-like emission is observed. The solvent polarity has a significant effect on the excimer contribution to the overall emission spectrum. The largest contribution of the excimer-like emissions is observed in the case of cyclohexane. The studied polymers have temperature-dependent fluorescence that can be utilized in the development of fluorescent temperature sensors. The time-resolved fluorescence measurements reveal that in the excited states, the polymer chains seem to hinder formation of excimers with equilibrium geometry. Apparently, a variety of excimers with different geometry are responsible for the observed fluorescence. Thermally-activated delayed fluorescence was observed for both samples in the solution and the solid state. The spectra of delayed fluorescence can be controlled by a fluorophore concentration and a solvent nature. Thus, the prepared polymers possess intriguing properties that make them very promising materials for use in optical, sensor, and imaging applications.

## Figures and Tables

**Table 1:** Thermal properties of polymers **1 a,b** and **3 a,b**.

Compound	T_d_^5% *a*^ (°C)		Residue after Decomp in Air (%)		T_g_ *^b,c^* (°C)	T_cc_ (°C)	ΔH_cc_ (J/g)	T_m_ (°C)	ΔH_m_ (J/g)
	in Air	in Argon	in Air	in Argon					
**1 a**	382	398	17	2	n	n	n	n	n
**1 b**	383	404	10	0	n	n	n	n	n
**3 a**	323	347	19	4	−121	-	-	-	-
**3 b**	393	413	5	4	−125	−95	12	−44	25

*^a^* T_d_^5%^—the temperature at which a weight loss of 5% was detected. *^b^* T_g_—the glass transition temperature. *^c^* DSC analysis was not performed for samples **1 a,b**.

**Table 2:** Optical properties of polymers **3 a,b** and model compound **4**.

Compound	Solvent	λ_abs_ (nm)	ε (M^−1^ cm^−1^)	λ_em_ *^a^* (nm)	Φ_f_ *^b^*	I_ex_/I_m_ *^c^*
**3 a**	Cyclohexane	379	-	406, 426 (m) 550 (e)	0.28	2.28
	Toluene	401, 385	-	424 (m) 550 (e)	0.44	0.30
	Dichloromethane	403, 387	-	430 (m) 550 (e)	0.60	0.12
**3 b**	Cyclohexane	394, 376	-	405, 425 (m) 550 (e)	0.50	0.06
	Toluene	401, 383	-	422 (m)	0.73	0.02
	Dichloromethane	402, 386	-	429 (m)	0.73	0.01
**4**	Cyclohexane	394, 376	60,700	405, 425 (m)	0.82	-
	Toluene	402, 385	54,400	422 (m)	0.87	-
	Dichloromethane	403, 388	65,200	430 (m)	0.87	-

λ_abs_—Absorption wavelength; λ_em_—emission wavelength; ε—extinction coefficient at the maximum; Φ_f_—fluorescence quantum yield. *^a^* m—monomer, e—excimer. *^b^* A solution of 9,10-diphenylanthracene in cyclohexane was used as a standard for calculating the quantum yield (Φ_f_ = 0.9). *^c^* The spectra were normalized at the maximum of monomer emission. Excimer intensity was measured at 540 nm. I_ex_/I_m_ was calculated using equation: I_ex_/I_m_ = (I_ex540_ − I_Mono-DBMBF2540_)/I_norm_, where I_ex_ is the intensity of excimer emission; I_m_ denotes the intensity of monomer emission; I_ex540_ is the intensity at 540 nm in normalized emission spectra of **3 a,b** (540 nm was selected as the wavelength close to the excimer emission maximum); I_Mono-DBMBF2540_ denotes intensity at 540 nm in normalized emission spectra of **4**; I_norm_ is the intensity used for the normalization of emission spectra at the maximum.

**Table 3:** I_ex_/I_m_ ratio in cyclohexane and toluene solutions of polymer **3 a** at different temperatures.

Compound	Solvent	T (°C)	I_ex_/I_m_
**3 a**	Cyclohexane	0	2.335
		10	2.068
		20	1.788
		30	1.55
		40	1.389
		50	1.276
		60	1.2
	Toluene	0	0.278
		10	0.276
		20	0.269
		30	0.262
		40	0.254
		50	0.25
		60	0.245
		70	0.244
		80	0.244

**Table 4:** Global fitting results obtained for fluorescence decays of polymers **3 a,b** in various solvents, λ_ex_ = 375 nm.

Polymer	Solvent	τ_1_ (ns)	τ_2_ (ns)	τ_3_ (ns)	τ_4_ (ns)
**3 a**	Cyclohexane	0.16	1.1	6.18	56.0
	Toluene	0.52	1.39	10.9	48.5
	Dichloromethane	0.23	1.26	3.53	52.5
**3 b**	Cyclohexane	0.27	1.45	7.65	55.3
	Toluene	0.44	1.84	10.85	54
	Dichloromethane	0.18	1.7	6.5	51.1

**Table 5:** Results of multiexponential fitting obtained for delayed fluorescence kinetics of polymers **3 a,b** at 540 nm in various deaerated solvents and the deaerated solid state (λ_ex_ = 375 nm).

Polymer	Solvent	τ (μs)	A (%)
**3 a**	Solid state	15,800	15
		5600	28
		570	57
	Cyclohexane	44	16
		9	84
	Toluene	51	13
		8	88
	Dichloromethane	68	57
		14	43
**3 b**	Solid state	15,800	38
		4000	62
	Cyclohexane	142	30
		31	70
	Toluene	96	33
		15	67
	Dichloromethane	64	20
		10	80

## Data Availability

The data presented in this study are available on request from the corresponding author.

## Supplementary Materials

The following supporting information can be downloaded at: https://www.mdpi.com/article/10.3390/polym14235075/s1.
