# Supplementary material for: Polydimethylsiloxanes with Grafted Dibenzoylmethanatoboron Difluoride: Synthesis and Properties
Source: Polymers (Basel). 2022 Nov 23;14(23):5075. doi: 10.3390/polym14235075 (PMC9740564; doi:10.3390/polym14235075)
Supplement: Supplementary file 1 [file polymers-14-05075-s001.zip › polymers-2038950-supplementary.pdf]

Supporting Information

Polydimethylsiloxanes with grafted dibenzoylmethanatoboron difluoride: synthesis and properties

Anastasia S. Belova,[1] Arevik G. Khchoyan,[1,2] Tatiana M. Il'ina,[1,2] Yuriy N. Kononevich,[1,*] Dmitry S. Ionov,[3] Viacheslav A. Sazhnikov,[3] Dmitry A. Khanin,[1] Galina G. Nikiforova,[1] Viktor G. Vasil'ev,[1] and Aziz M. Muzafarov[1,4]

[1] A.N. Nesmeyanov Institute of Organoelement Compounds of Russian Academy of Sciences, 119991 Moscow, Russian Federation

[2] D. Mendeleev University of Chemical Technology of Russia, 125047 Moscow, Russian Federation

[3] Photochemistry Center, FSRC "Crystallography and Photonics" of Russian Academy of Sciences, 1119421 Moscow, Russian Federation

[4] N.S. Enikolopov Institute of Synthetic Polymeric Materials of Russian Academy of Sciences, 117393 Moscow, Russian Federation

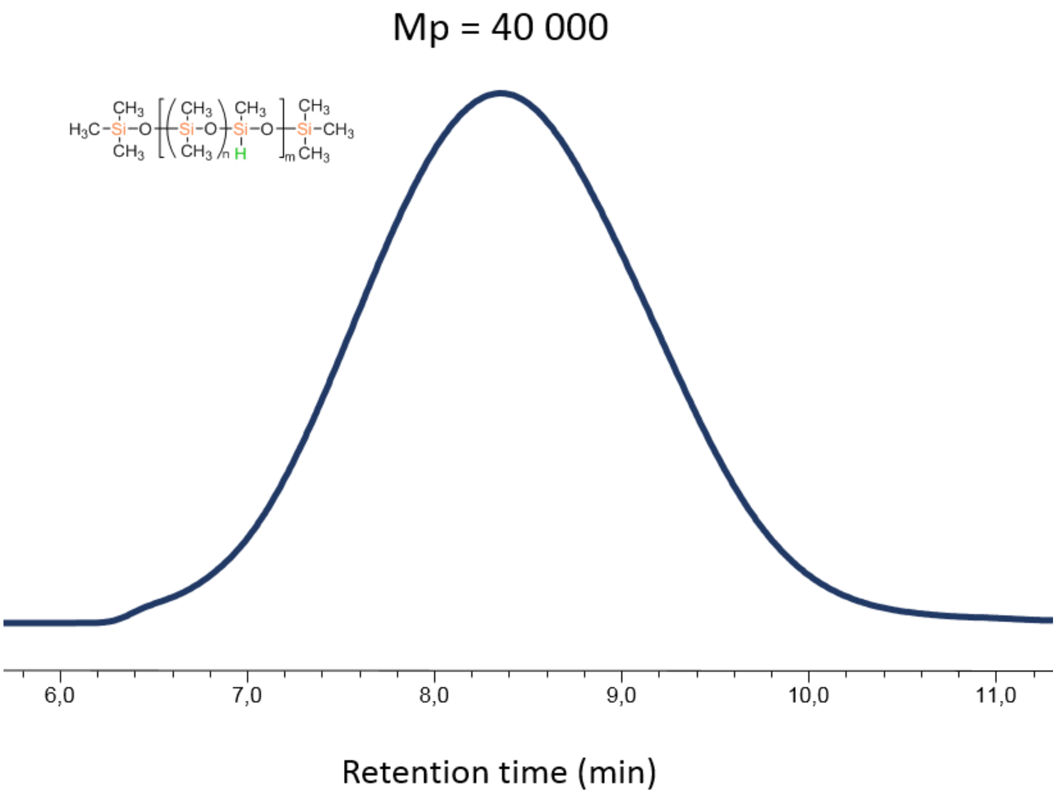

Figure S1. GPC curve of polymer **1 a** obtained in toluene using column Phenogel 10^4 Å.

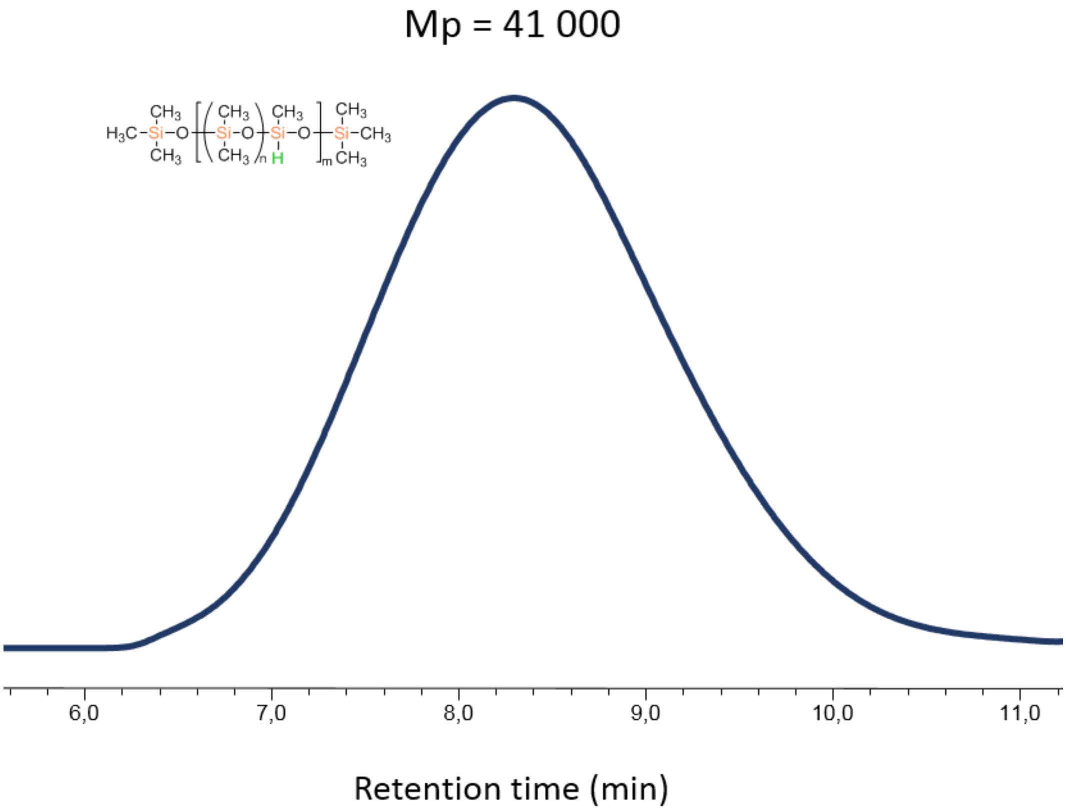

Figure S2. GPC curve of polymer **1 a** obtained in toluene using column Phenogel 10^4 Å.

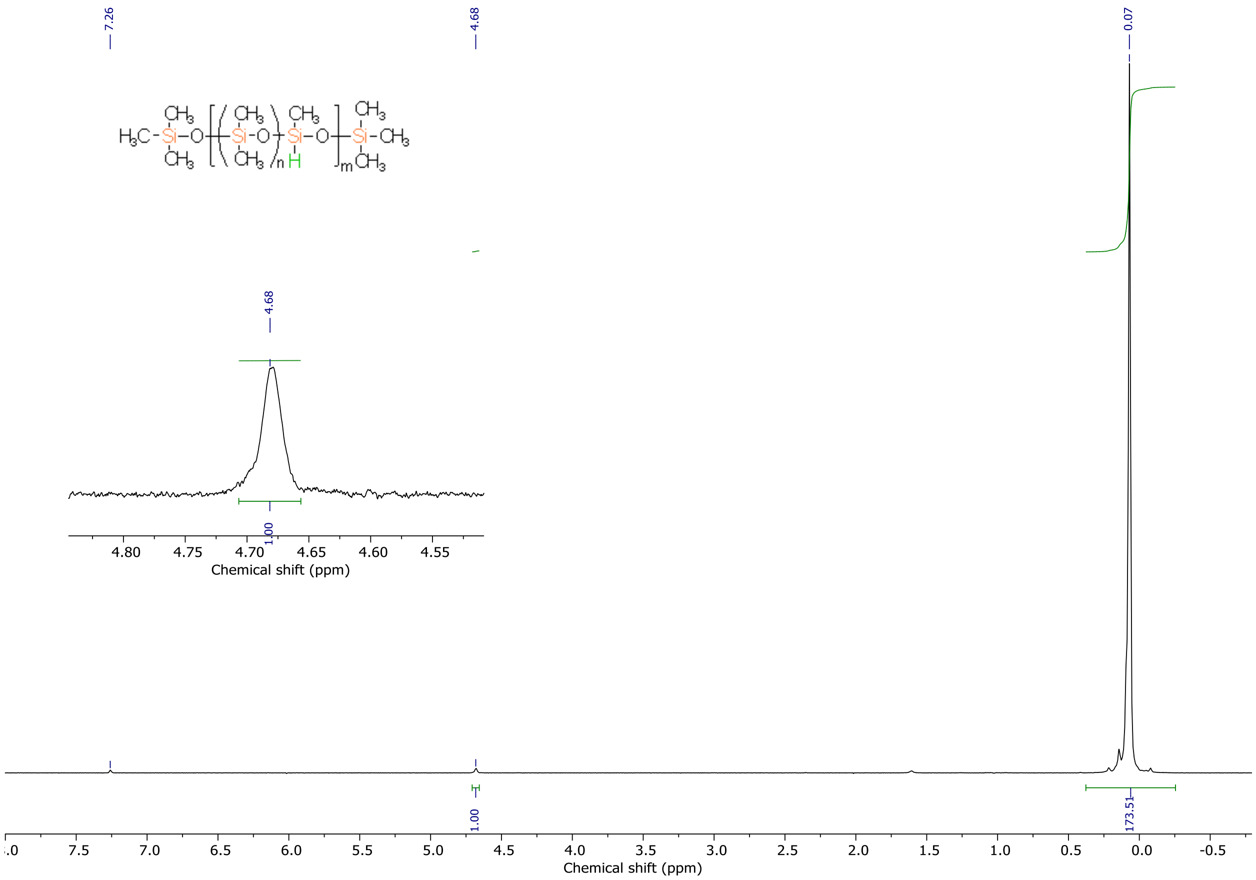

Figure S3. ^{1}H NMR spectrum of polymer **1 a** in $CDCl_3$.

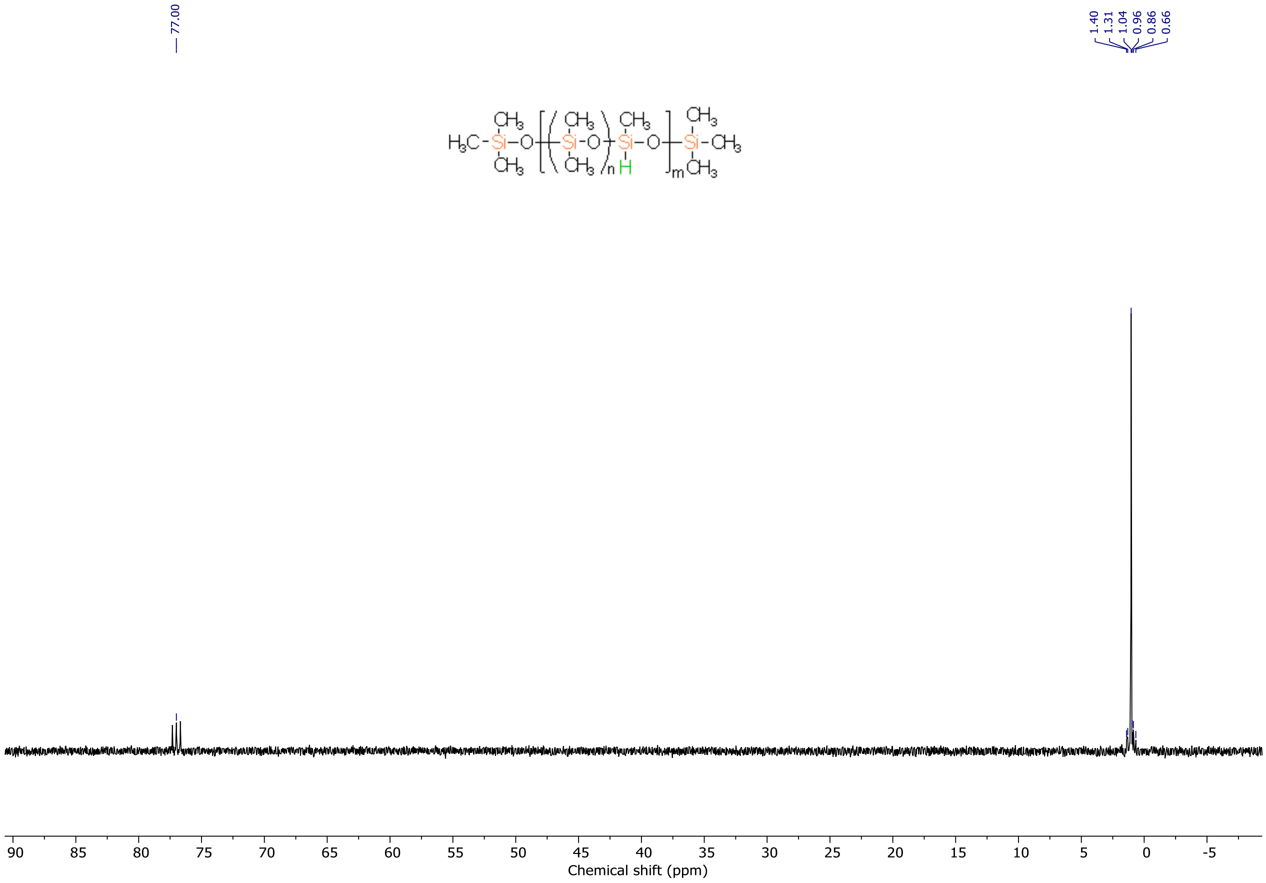

Figure S4. ^{13}C NMR spectrum of polymer **1 a** in $CDCl_3$.

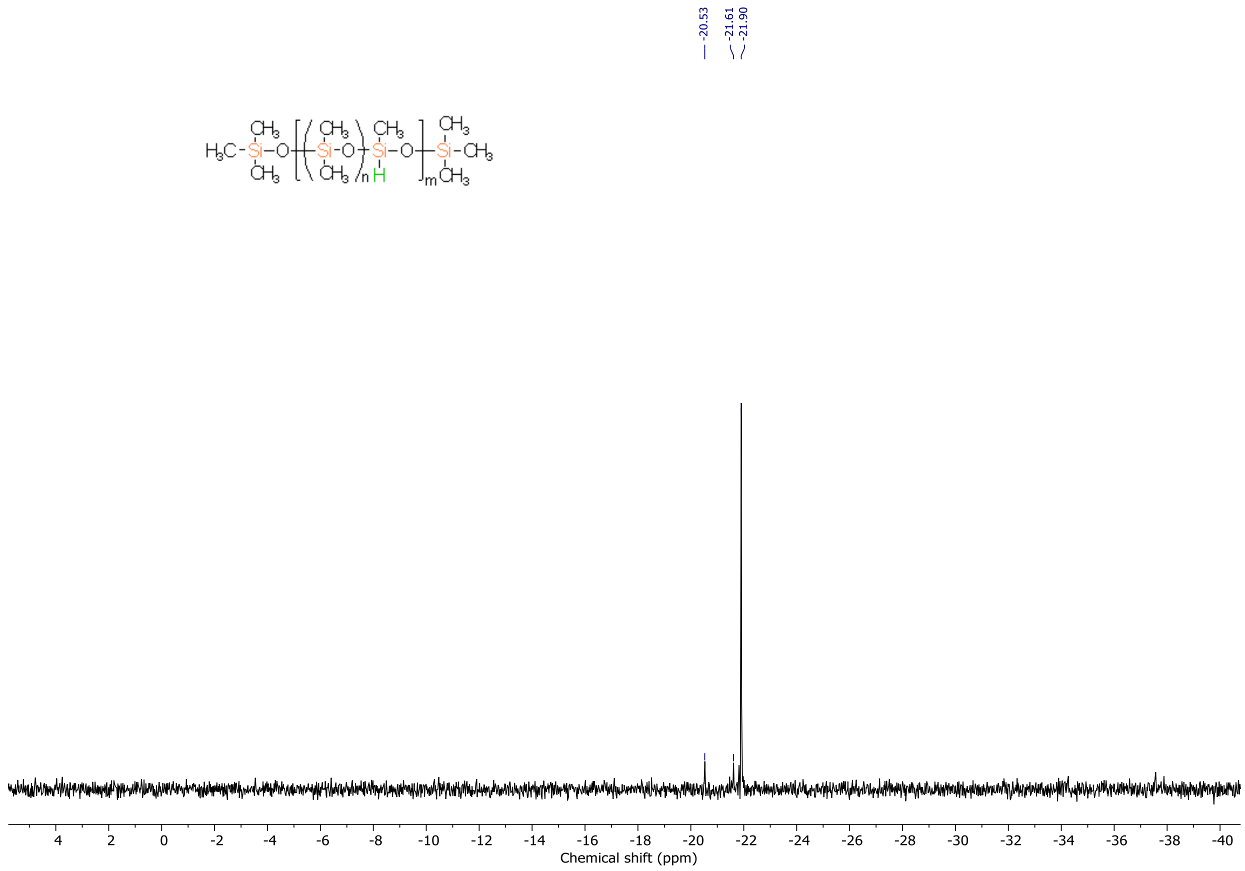

Figure S5. ^{29}Si NMR spectrum of polymer **1 a** in $CDCl_3$.

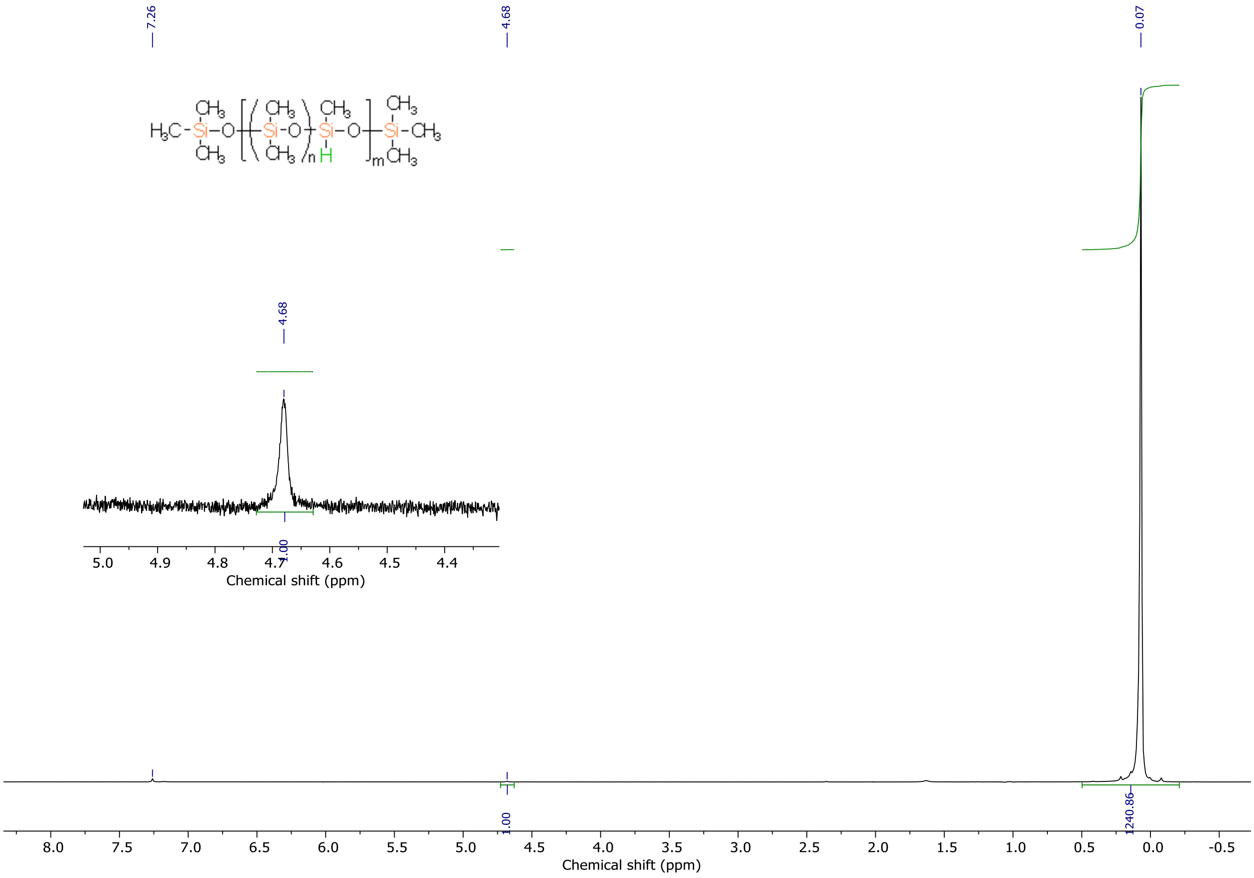

Figure S6. ^{1}H NMR spectrum of polymer **1 b** in $CDCl_3$.

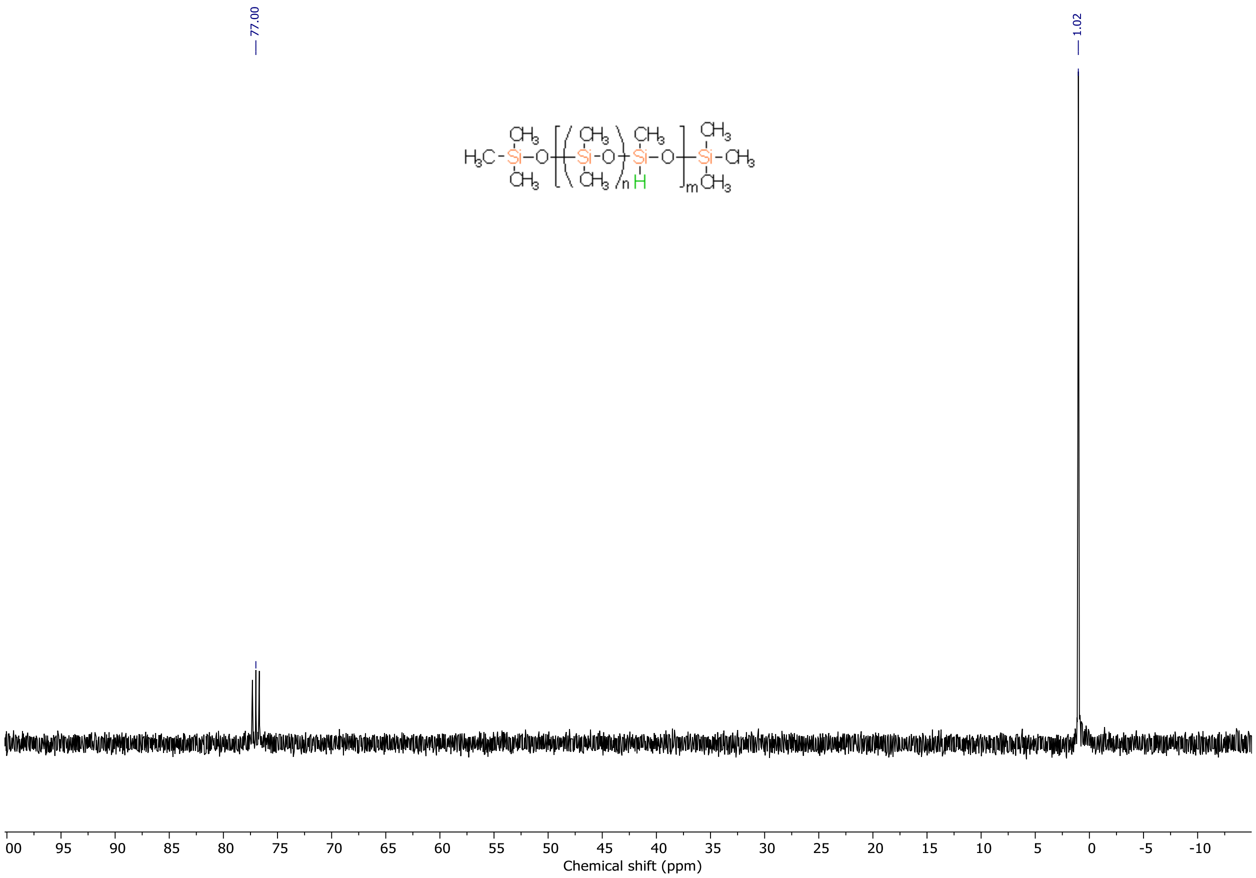

Figure S7. ^{13}C NMR spectrum of polymer **1 b** in $CDCl_3$.

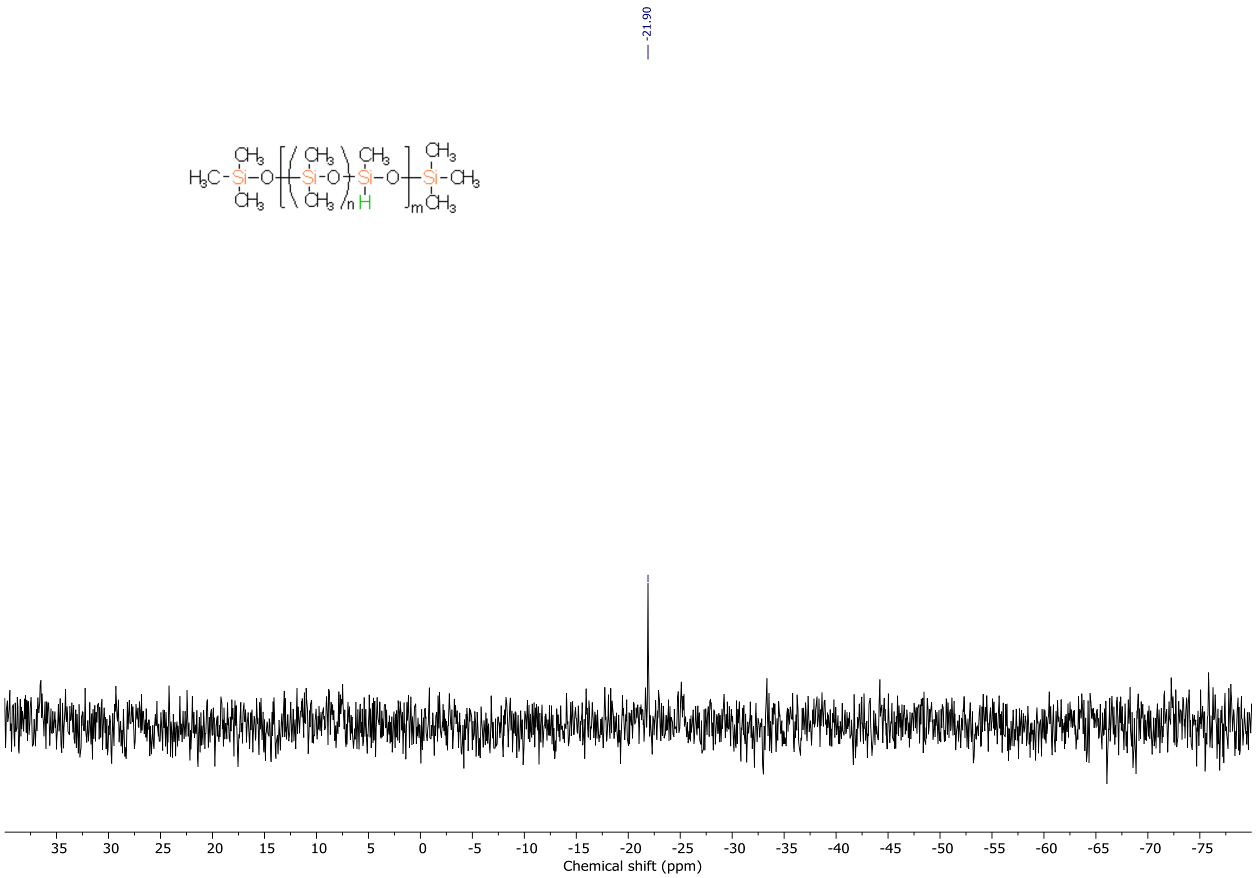

Figure S8. ^{29}Si NMR spectrum of polymer **1 b** in $CDCl_3$.

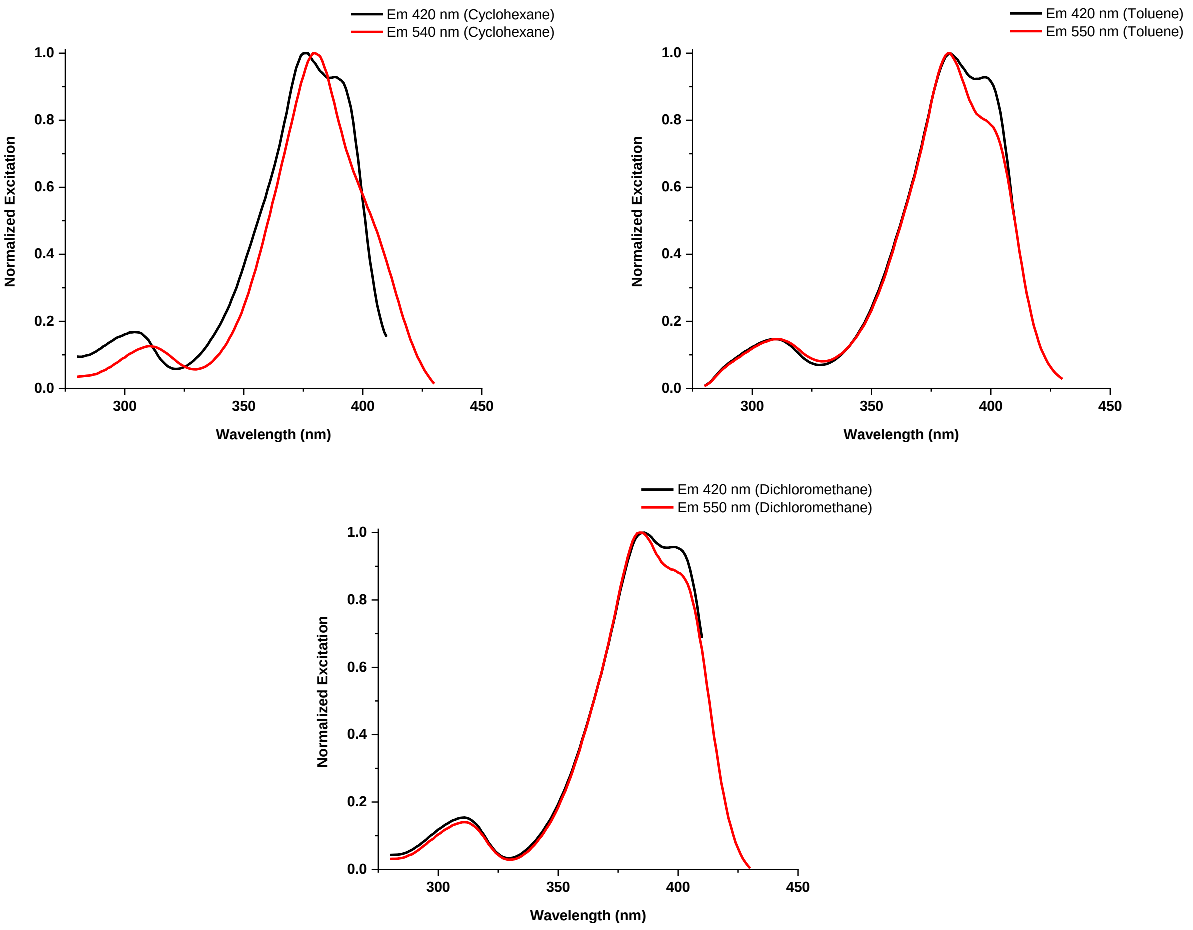

Figure S9. Normalized fluorescence excitation spectra of polymer **3 a** in various solvents (RT, OD<0.1).

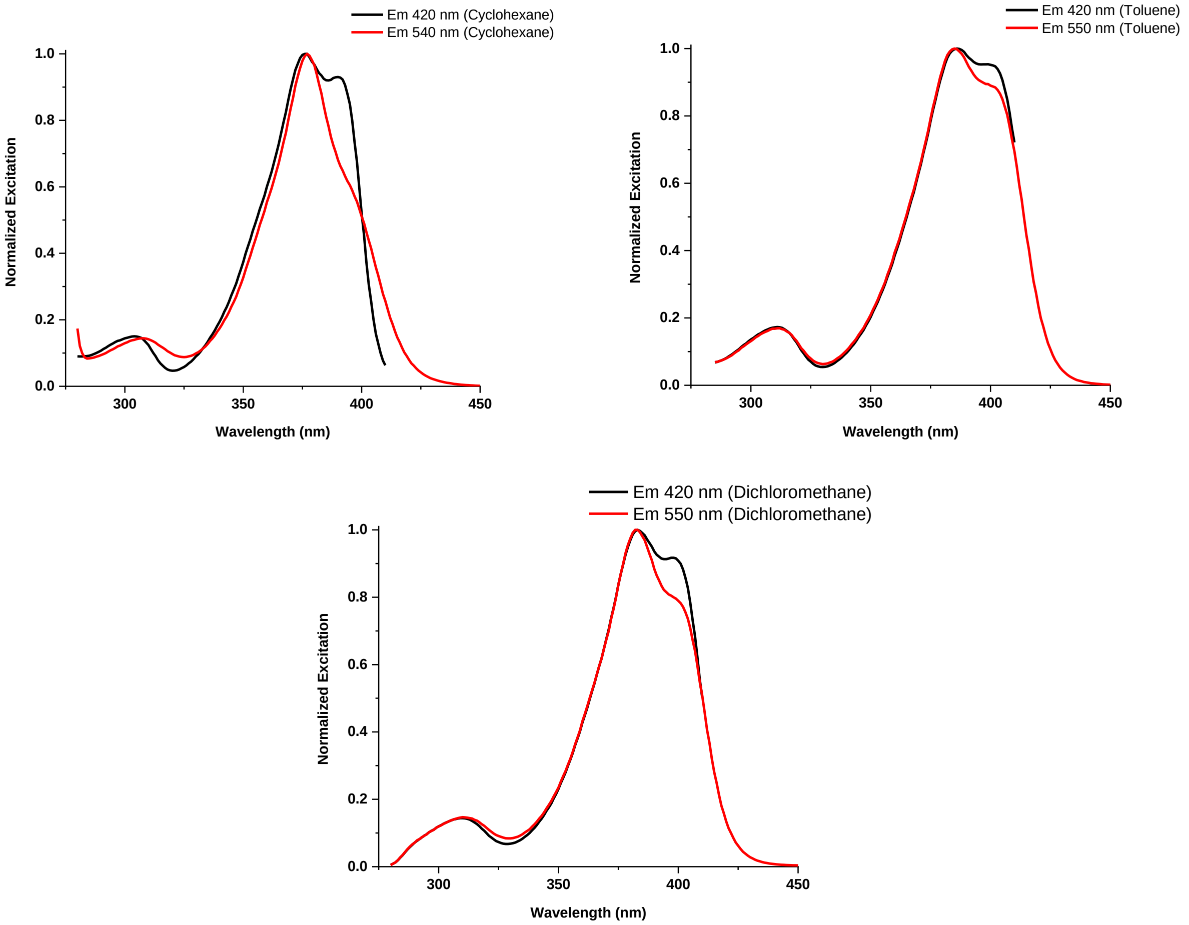

Figure S10. Normalized fluorescence excitation spectra of polymer **3 b** in various solvents (RT, OD<0.1).

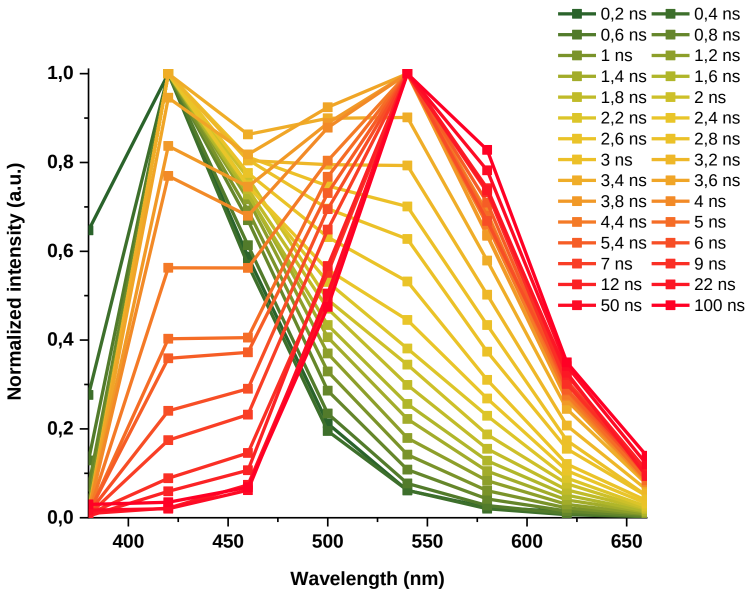

Figure S11. The fluorescence spectra obtained from the fluorescence kinetics in Figure 30 at various times in the range of 0.2-100 ns normalized to maximum at a given time.

Table S1. Results of multiexponential fitting obtained for fluorescence decays of polymers **3 a,b** at 436 and 540 nm in various solvents (λ_{ex} = 375 nm).

**Polymer**	**Solvent**	**436 nm**		**540 nm**	
		**τ (ns)**	**A (%)**	**τ (ns)**	**A(%)**
**3 a**	Cyclohexane	1.48	15	55.2	51
		0.66	28	2.36	49
		0.14	57		
	Toluene	1.85	11	47.3	22
		0.99	51	1.33	78
		0.25	37		
	Dichloromethane	2.63	5	53.9	42
		1.22	65	7.42	16
		0.25	30	1.53	42
**3b**	Cyclohexane	1.45	67	55.3	24
		0.32	33	1.59	76
	Toluene	1.79	78	51.8	4
		0.30	22	1.87	96
	Dichloromethane	1.76	81	47.1	3
		0.49	19	1.87	97

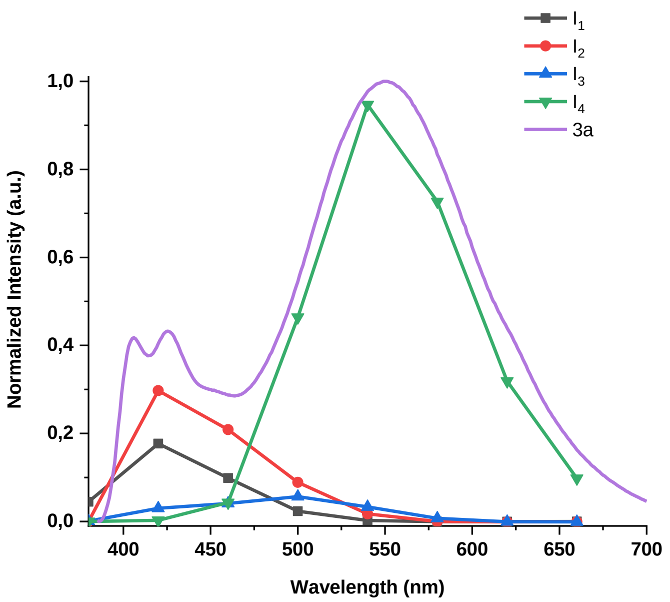

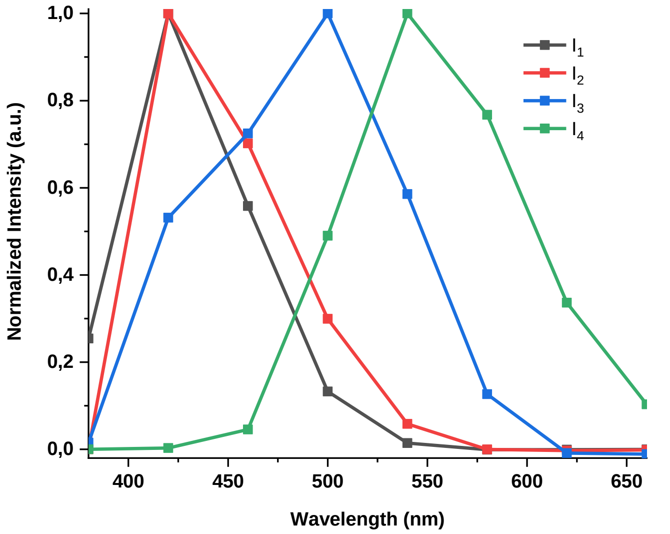

Figure S12. $I_1 - I_4$ intensities spectra corresponding to the exponential terms with lifetimes τ_{1}- τ_4 obtained from the global fitting of fluorescence decays of **3 a** in cyclohexane and comparison with steady-state fluorescence spectra for **3 a** (top); normalized spectra of $I_1 - I_4$ intensities for **3 a** (bottom) in cyclohexane.

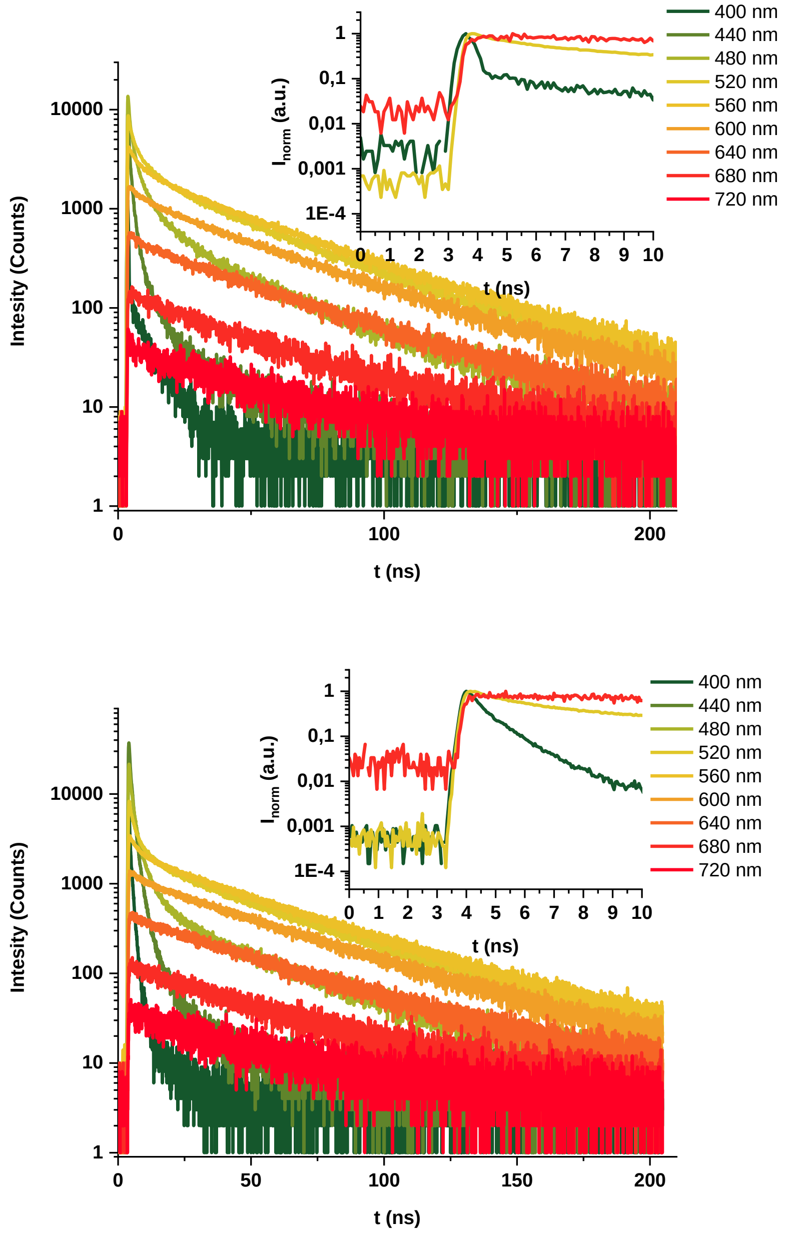

Figure S13. Fluorescence decay curves for polymers **3 a** (top) and **3 b** (bottom) at series of wavelengths in the range of 380-660 nm in the solid state (λ_{ex} = 375 nm). In the insets the comparison of normalized decays obtained at 300, 520, 680 nm is shown.

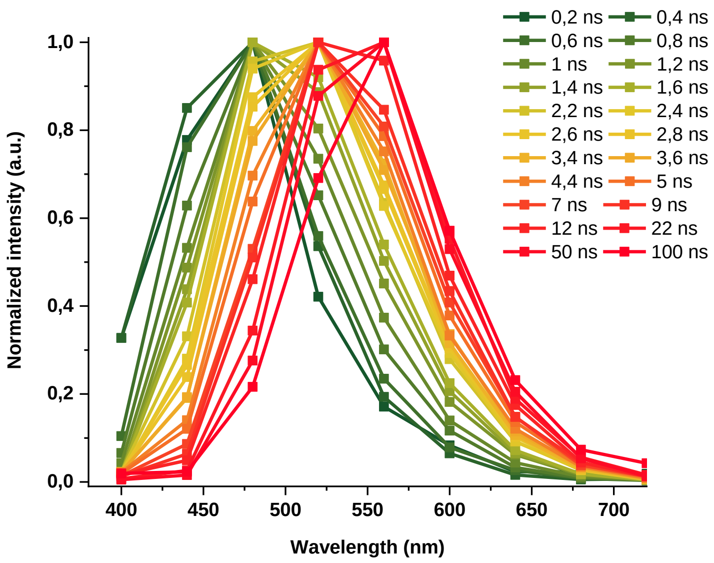

Figure S14. The fluorescence spectra of **3 a** in the solid state obtained from the fluorescence kinetics in Figure 33 at various times in the range of 0.2-100 ns normalized to maximum at a given time.

Table S2. Global fitting results obtained for fluorescence decays of **3 a,b** in the solid state, λ_{ex} = 375 nm.

**Polymer**	**τ_1 (ns)**	**τ_2 (ns)**	**τ_3 (ns)**	**τ_4 (ns)**	**τ_5 (ns)**
**3 a**	0.323	1.55	6.46	27	53.54
**3 b**	0.397	1.412	4.2	17.6	47.86

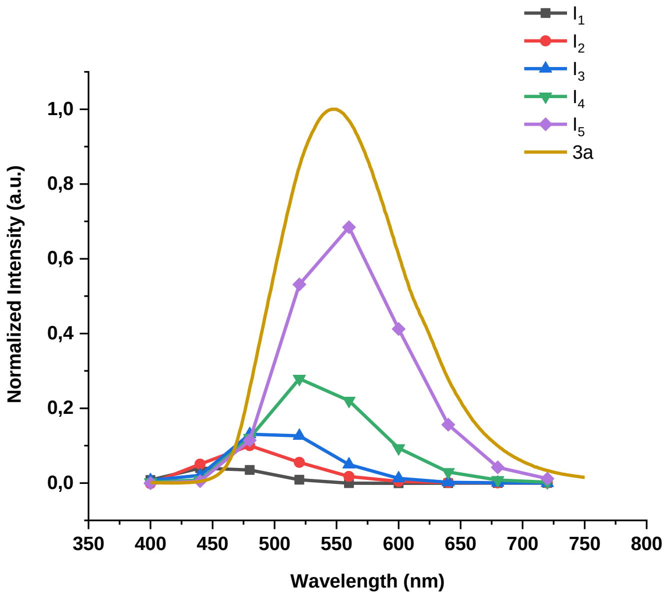

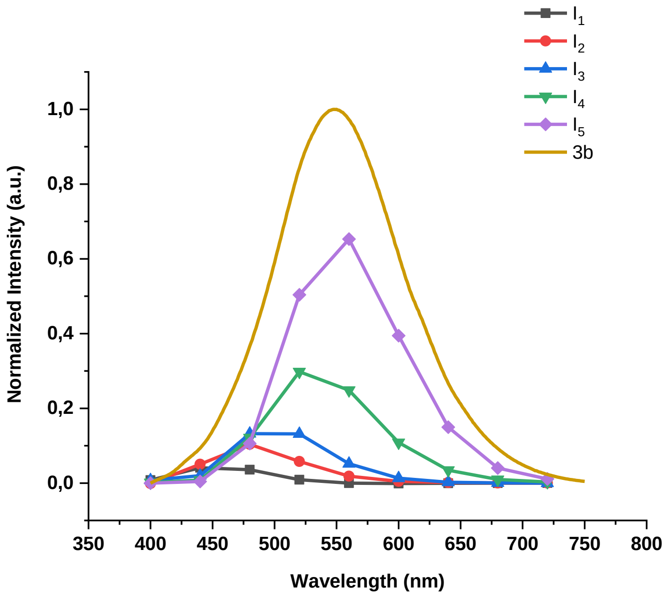

Figure S15. $I_1 – I_4$ intensities spectra corresponding to the exponential terms with lifetimes τ_{1}- τ_5 obtained from the global fitting of fluorescence decays of **3 a** (top) and **3 b** (bottom) in the solid state and comparison with steady-state fluorescence spectra.
